# The PACE Trial’s GET Manual for Therapists Exposes the Fixed Incremental Nature of Graded Exercise Therapy for ME/CFS

**DOI:** 10.3390/life15040584

**Published:** 2025-04-02

**Authors:** Mark Vink, Katarzyna Partyka-Vink

**Affiliations:** 1 Family and Insurance Physician, Independent Researcher, 1096 HZ Amsterdam, The Netherlands; 2 Independent Researcher, 49032 Osnabrück, Germany

**Keywords:** CFS, chronic fatigue syndrome, eminence-based medicine, evidence-based medicine, ME, NICE, post-infectious disease

## Abstract

The British National Institute for Health and Care Excellence (NICE) published its updated guidelines for myalgic encephalomyelitis/chronic fatigue syndrome (ME/CFS) in October 2021. NICE concluded, after an extensive review of the literature, that graded exercise therapy (GET) is harmful and should not be used, and that cognitive behavioural therapy (CBT) is only an adjunctive and not a curative treatment. An article by White et al., which is written by 51 researchers, claims that there are eight anomalies in the review process and the interpretation of the evidence by NICE. In this article, we reviewed the evidence they used to support their claims. Their three most important claims are that NICE redefined the disease, that CBT and GET are effective, and that fixed incremental increases are not part of GET. However, our analysis shows that the disease was not redefined by NICE. Instead, it was redefined in the 1990s by a group of doctors, including a number of authors of White et al., when they erased the main characteristic of the disease (an abnormally delayed muscle recovery after trivial exertion, which, over the years, has evolved into post-exertional malaise) and replaced it with chronic disabling severe fatigue. Their own studies show that CBT and GET do not lead to a substantial improvement of the quality-of-life scores or a reduction in CFS symptom count, nor do they lead to objective improvement. Also, both treatments have a negative instead of a positive effect on work and disability status. Moreover, a recent systematic review, which included one of the authors of White et al., showed that ME/CFS patients remain severely disabled after treatment with CBT. Our analysis of, for example, the PACE trial’s GET manual for therapists exposes the fixed incremental nature of GET. Why the authors are not aware of that is unclear because eight of them were involved in the PACE trial. Three of them were centre leaders and its principal investigators, four others were also centre leaders, and another one was one of the three independent safety assessors of the trial. Moreover, many of these eight authors wrote, or were involved in writing, this manual. In conclusion, our analysis shows that the arguments that are used to claim that there are eight anomalies in the review process and the interpretation of the evidence by NICE are anomalous and highlight the absence of evidence for the claims that are made. Furthermore, our analysis not only exposes the fixed incremental nature of GET, but also of CBT for ME/CFS.

## 1. Introduction

The British National Institute for Health and Care Excellence (NICE) published its updated guidelines on the diagnosis and management of myalgic encephalomyelitis/chronic fatigue syndrome (ME/CFS) in October 2021 [1]. NICE concluded that ME/CFS is a complex multisystem chronic medical condition [1]. The prestigious American Institute of Medicine (IOM, now the National Academy of Medicine), the Dutch Health Council, the Superior Health Council of Belgium and the German independent Institute for Quality and Efficiency in Healthcare (Institut für Qualität und Wirtschaftlichkeit im Gesundheitswesen, IQWiG) came to a similar conclusion in 2015, 2018, 2020 and 2021, respectively [2,3,4,5].

NICE also concluded that cognitive behavioural therapy (CBT) and graded exercise therapy (GET) do not lead to improvement or recovery. CBT should only be offered as a supportive therapy and GET, or other programs based on fixed incremental increases in physical activity or exercise, are harmful and should not be offered for the treatment of ME/CFS. Also, NICE emphasised that people should remain in their energy envelope when undertaking activity of any kind [1]. These conclusions are in line, for example, with the conclusions from the IOM, which concluded that there are no effective treatments for ME/CFS [2], and the German IQWiG [5], which reports that there is a risk of serious side-effects when GET is used, although according to IQWiG, it is unclear if this is caused by the treatment or the incorrect use of it (in German: “das Risiko für schwerwiegende Nebenwirkungen … wurde dieses Risiko in der Anhörung zum Vorbericht herausgestellt, wobei unklar blieb, ob sich diese Berichte auf eine nicht sachgemäße Anwendung der GET beziehen” (p. v [5]). The IQWiG also concluded that pacing is one of the most important instruments that patients have to live and deal with their symptoms (in German: “Energiemanagement (Pacing)…stellt eines der wichtigsten Instrumente dar, das Patientinnen und Patienten mit ME/CFS zur Verfügung steht, um sie dabei zu unterstützen, mit den Symptomen ihrer Erkrankung zu leben” (p. 51 [5]).

Worldwide proponents of the cognitive behavioural model (CBmodel) [6] recently stated in an article entitled Anomalies in the review process and interpretation of the evidence in the NICE guideline for chronic fatigue syndrome and myalgic encephalomyelitis that “this U-turn in recommendations from the previous 2007 guideline is controversial. We suggest that the controversy stems from anomalies in both processing and interpretation of the evidence by the NICE committee” (p. 1 [6]). They then list eight different anomalies and “conclude that the dissonance between this and the previous guideline was the result of deviating from usual scientific standards of the NICE process. The consequences of this are that patients may be denied helpful treatments and therefore risk persistent ill health and disability” (p. 1 [6]).

In this article, we will review the evidence presented in that article by White et al. [6] in support of the aforementioned eight anomalies, to see if there is any merit in it and if NICE should have come to different conclusions. This is especially important because, according to a conservative estimation, there are at least 400 million suffering from post-COVID syndrome [7], which is more commonly referred to as long COVID, after an initial, often mild, infection with SARS-CoV-2. According to a systematic review, 51% of long COVID patients fulfil ME/CFS criteria [8]. Put differently, 51% of people with long COVID have ME/CFS which has been triggered by SARS-CoV-2. If White et al. are right, then there would be effective treatments for those 51% (more than 200 million patients), as well as the estimated 17 to 24 million people with ME/CFS which were already afflicted before the SARS-CoV-2 epidemic [9]. If, on the other hand, NICE is right, then that would mean that there are no effective treatments for around 420 million people with long COVID and ME/CFS. This not only constitutes a health crisis for the people involved, but it also constitutes an economic crisis because most of those patients are between 20 and 50 and in the prime of their economic and productive life. The economic costs of long COVID disabling a previously productive workforce are significant. For example, estimates place the total US economic cost of long COVID in the range of USD 140 to USD 600 billion per year [10]. This makes it even more important to find out if CBT and GET are effective treatments, as claimed by White et al., or if they do not lead to improvement and recovery, as concluded by NICE. If the latter is the case, then there is an urgent need for causative effective treatments approaches for the more than 400 million people with post-infectious diseases [11].

## 2. The Uncontroversial Conclusions About CFS/ME in the Guideline According to White et al.

White et al. [6] state in their article that there are eight uncontroversial conclusions in the guideline, which include:

- “CFS/ME is a serious and debilitating condition.
- Some patients are severely disabled, which may limit access to care and treatment.
- Postexertional malaise is a common and important symptom of the illness.
- CFS/ME shows pathophysiological changes, but there are no diagnostic tests.”
- “Treatments for CFS/ME should be negotiated between healthcare professionals and patients and should always be delivered collaboratively.”
- “Evidenced-based therapies for CFS/ME, such as cognitive behavioural therapy and graded exercise therapy, do not benefit all patients” (p. 2 [6]).

In regard to the first point, the basis of the cognitive behavioural model (CBmodel) upon which CBT and GET are based is that, after a viral illness, patients continue to avoid exercise, demonstrating false beliefs that there is an underlying debilitating illness, which leads to deconditioning. The more they avoid exercise, the worse the deconditioning becomes. According to this model, there is no underlying illness, and symptoms in ME/CFS are caused by the deconditioning. CBT, which contains an element of graded exercise therapy (graded activity), and GET are designed to reverse that and lead to recovery. The CBmodel [12,13,14] was created by a number of authors of White et al. (Wessely, David, Chalder, Sharpe and van der Meer) [6] in the 1990s. The CBmodel is the reason that the medical world sees ME/CFS as a psychosomatic disease and that many doctors do not believe in this disease. If the authors now state that it is uncontroversial that ME/CFS is a serious and debilitating condition, does that mean that the authors are now acknowledging the fact that there is an underlying illness and that their CBmodel is incorrect? Also, as we will see under point 3.1, post-exertional malaise (PEM) is not a common and important symptom of the illness, but it is the defining characteristic.

If the guideline would have concluded that “evidenced-based therapies for CFS/ME, such as cognitive behavioural therapy and graded exercise therapy, do not benefit all patients” (p. 2 [6]), which implies that it benefits most patients, then there would have been no need for White et al. to write an article claiming that there are anomalies in the review process and the interpretation of the evidence by NICE because the NICE ME/CFS guideline would then recommend treatments upon which many of the authors of White et al. have based their careers. In reality, the guideline concluded that both treatments do not lead to improvement or recovery and both treatments should therefore not be used. Moreover, it also concluded that GET should not be used because it is harmful [1].

As far as “some patients are severely disabled, which may limit access to care and treatment” (p. 2 [6]) are concerned, 25% of patients are severely or very severely affected and many of them are bedridden and dependent on others [15]. Many of them do not have any access to care and treatment because many doctors refuse to see and treat these patients. This is based on the aforementioned CBmodel and its assumption that ME/CFS is a psychosomatic illness which has been promoted by authors of White et al. who have successfully built a career on it. The consequence of this is a paradox of care in that the more severely disabled ME/CFS patients are, the less care they receive from the medical profession. Moreover, as concluded by NICE and the IOM, there is no effective treatment for ME/CFS [1,2]. Consequently, it is not the severity of the disease which limits access to treatment. It is the attitude of the medical profession and the absence of effective treatment that limits it.

White et al. [6] also state that “treatments for CFS/ME should be negotiated between healthcare professionals and patients and should always be delivered collaboratively” (p. 2 [6]). Yet, as noted by Edwards, “there is nothing to be ‘negotiated’ about treatments. The job of the healthcare professional is to offer what they believe are useful treatments, based on evidence, no more. The decision to take up a treatment is ENTIRELY a matter for the patient. There is absolutely nothing to ‘negotiate’. The healthcare professionals are paid to provide a service. The patient has no obligation to offer anything in exchange—which is what negotiation means. This is an admission of a totally outdated and inappropriate view of delivering healthcare. And nothing is to be ‘delivered collaboratively’. It is to be delivered competently by the professional. The whole concept of ‘shared decision making’ is in reality the opposite of what it is billed as. It is just a softer form of coercion” [16].

Finally, there is an important omission in that list because there are many scientific papers which have provided proof that exercise leads to all sorts of physiological abnormalities, which is why any form of exercise treatment is so harmful for ME/CFS patients [17,18,19,20,21,22,23,24,25,26,27,28,29]. For example, Kujawski et al. specifically looked at the effects of exercise in ME/CFS and they found that “exercise was not well tolerated by 51% of patients” (p. 1 [19]).

## 3. The Eight Anomalies According to White et al.

### 3.1. Use of a New Definition of CFS/ME Downgraded the Certainty of Trial Evidence

#### 3.1.1. Post-Exertional Malaise (PEM)

White et al. [6] state in their article that “while there is strong evidence that PEM is an important and common symptom of CFS/ME, the new guideline made it mandatory for making the diagnosis. This is problematic as PEM is not a mandatory symptom in the Centers for Disease Control and Prevention (CDC) definition which, with over 6000 citations on Google Scholar, is far and away the most widely researched definition of the condition” (p. 3 [6]). The name myalgic encephalomyelitis (ME) goes back to an outbreak in the Royal Free hospital in London in 1955. This outbreak was witnessed and documented by Doctor Melvin Ramsay, the infectious disease specialist from the hospital. He documented that the main characteristic of the disease was an abnormally delayed muscle recovery after trivial exertion which, over time, evolved into PEM [30]. He also documented that “without it I would be unwilling to diagnose a patient as suffering from ME” (p. 1 [31]).

#### 3.1.2. Erasing the Main Characteristic of the Disease

In 1991, a group of British doctors, which included White and three other authors of White et al. (David, Sharpe and Wessely), created the Oxford criteria. The aforementioned main characteristic of ME/CFS was erased and replaced by chronic disabling severe fatigue that “should have been present for a minimum of 6 months during which it was present for more than 50% of the time” (p. 119 [32]). By doing so they redefined the disease. It also means that they turned it into a part-time disease because patients could be well for up to 49% of the time. Five authors of White et al. (Abbey, Buchwald, Lloyd, Sharpe and Wessely) [6] were involved in creating the aforementioned CDC definition, more commonly known as the Fukuda criteria from 1994 [33]. According to this definition, PEM is only an optional criterion and is not required for diagnosis either. Consequently, ME/CFS was redefined in 1991 and in 1994 involving authors of White et al. [6]. Moreover, an additional problem of these criteria is that they do not provide severity classes and in general are not very usable to study progression or recovery of the disease.

As noted by a review of case definitions by Christley et al., “the pre-eminence of this definition [the Fukuda definition] over the others has never been substantiated and it has been widely criticised for its lack of specificity” (p. 25 [34]). A systematic review by Ahmed et al. noted that “the Oxford and CDC criteria have been criticized for focusing on fatigue in general and for not including PEM, a key symptom of ME/CFS” (p. 13 [35]). Professor Hooper wrote an article about redefining ME/CFS by creating the Oxford criteria, entitled *Magical Medicine: How to make a disease disappear* [36], in which he shows that ME/CFS was redefined in such a way that doctors now erroneously think that ME/CFS is being tired all the time.

But even if the CDC definition (the Fukuda criteria) would be the most widely researched definition of the condition, that does not mean that it is scientifically correct to use criteria that do not require the main characteristic of a disease to diagnose people with it. If patients do not suffer from PEM, then they do not have ME/CFS by definition.

The Agency for Healthcare Research and Quality (AHRQ) ME/CFS evidence report for the American National Institutes of Health was published in December 2014 and an addendum was published in 2016 [37]. This systematic review, carried out by Smith et al., concluded that post-exertional malaise is a critical component of ME/CFS (p. ES-1 [37]). They noted about the Oxford case definition that it “is the least specific of the definitions and less generalizable to the broader population of patients with ME/CFS” because “specific features of ME/CFS such as post-exertional malaise…a hallmark symptom of the disease” is not required for diagnosis. “As a result, using the Oxford case definition results in a high risk of including patients who may have an alternate fatiguing illness or whose illness resolves spontaneously with time”. Additionally, “the continued use of the Oxford…case definition ‘may impair progress and cause harm’”. Consequently, “future research should retire the use of the Oxford … case definition” (p. 1 [37]).

Why White et al. [6] have a problem with the fact that PEM is mandatory for diagnosis is unclear because one of their authors (Sharpe) wrote in 2002 that “chronic fatigue syndrome (CFS) describes a symptom-defined syndrome with fatigue, *typically exacerbated by exertion as the cardinal symptom*” [italic by us] (p. 427 [38]). Also, three of their authors (Sharpe, Chalder and White) submitted an article in August 2020, which was published in November 2021, in which they started the introduction in the following manner. “Chronic fatigue syndrome…is characterized by fatigue and other symptoms that are typically exacerbated by exertion (a phenomenon often referred to as post-exertional malaise or PEM)” (p. 449 [39]). Moreover, White et al. started the introduction of their current article in a similar way [6] and one of the two references at the end of that sentence refers to the report by the IOM from 2015 [2]. The IOM also noted that if patients do not suffer from PEM, then an alternative diagnosis should be considered [2].

#### 3.1.3. CBT and GET Are Ineffective, Irrespective of the Definition

Moreover, the IOM, as well as NICE and a number of reanalyses, did not ignore studies with case definitions that did not require PEM for diagnosis. Instead, they found that there is no evidence that CBT and/or GET are beneficial for ME/CFS patients on objective measures such as actigraphy, employment, illness and disability benefits, medical pensions, etc. *irrespective* of how ME/CFS is defined [1,2,35,40,41,42,43,44,45,46,47].

White et al. also state that “the emphasis NICE placed on PEM is debatable” because “it is not specific to CFS/ME, being found in many conditions which present with pathological fatigue” (p. 3 [6]). But why does a problem have to be unique or specific to one disease for it to be a specific problem? For example, shortness of breath is an important symptom of pneumonia but it is not specific to pneumonia as it is also an important symptom of many different forms of pulmonary and cardiovascular diseases. Additionally, any disease where patients suffer from PEM means that exercising your way out of the disease is impossible, be it ME/CFS, long COVID or other diseases [48].

#### 3.1.4. PEM Is Objectively Measurable

White et al. also state that “PEM is also subjective, by definition” (p. 3 [6]). However, a review of case definitions concluded in 2020 not only that “PEM [is] a key symptom of ME/CFS” but also that it is now possible to “objectively assess PEM” by using “a standardized technique to measure the level of oxygen uptake using cardiopulmonary exercise testing (CPET)” (p. 8 [49]). The first to objectively assess PEM was a group of exercise physiologists in 2010 who also noted that “postexertional malaise (PEM) is a defining characteristic of chronic fatigue syndrome” (p. 239 [23]).

Assessment of hand grip strength (HGS) is a highly reproducible tool to assess muscular strength, which provides information about the person’s physical function and state of health [50,51]. Maximal handgrip strength is significantly correlated with peak oxygen uptake and can predict maximal physical performance in CPET [51,52]. HGS is impaired in ME/CFS, and HGS studies have provided objective evidence for PEM and the abnormally delayed recovery in ME/CFS [51]. Additionally, Jäkel et al. assessed the HGS of 105 patients with ME/CFS in comparison to patients with cancer-related fatigue (CRF) and healthy controls (HCs). They concluded that repeat HGS assessment is a sensitive diagnostic test to assess muscular fatigue and fatigability and an objective measure to assess disease severity in ME/CFS [51].

White et al. state that NICE “downgraded nearly thirty years of research” if “trials…had not specifically and explicitly required participants to” have “PEM as a mandatory criterion for recruiting participants” (p. 3 [6]). Yet, the previous NICE ME/CFS guideline from 2007 already highlighted PEM as a core feature of ME/CFS when it stated that the fatigue in ME/CFS is “characterised by post-exertional malaise and/or fatigue (typically delayed, for example by at least 24 h, with slow recovery over several days)” (pp. 14–15 [53]). Moreover, the NICE guideline from 2007 also states that “the diagnosis of CFS/ME should be reconsidered if patients do not suffer from post-exertional fatigue or malaise” (pp. 18–19 [53]). Why this is unknown to White et al. is unclear because two of their authors (Hamilton and Santhouse) [6] were part of the guideline development group for the NICE ME/CFS guideline from 2007 (pp. 44–45 [53]).

#### 3.1.5. Redefining PEM by the PACE Trial

White et al. [6] state that the PACE trial showed that “PEM improved more with CBT and GET compared with the comparison treatments” (p. 3 [6]). Its three principal investigators, who were also centre leaders (Chalder, Sharpe and White), as well as four other centre leaders (Angus, Murphy, Wade and Wessely) and one doctor (Miller), who was one of the three independent assessors of the trial safety data [54], are all authors of White et al. [6]. The PACE trial, which was labelled as the “definitive randomised trial” (p. 3 [55]) by its investigators, is the largest CBT and GET trial ever conducted. According to the final version of the PACE trial protocol, PEM was defined as “feeling ill after exertion” (p. 156 [56]. However, PEM is characterised by a decrease in functioning and a worsening of ME/CFS symptoms after trivial physical or cognitive exertion and typically occurs with a time lag (hours or days) after physical or mental activity or stress with an abnormally delayed recovery [2]. Consequently, the PACE trial redefined PEM into something it is not.

#### 3.1.6. The GETSET Trial by White

White et al. also use the GETSET trial by White [57], the leading author of White et al. [6]. According to them, “one trial of self-help based on the principles of GET [the GETSET trial], which did use an illness definition that mandated PEM, found the exercise intervention was effective in reducing fatigue” (p. 3 [6]). The scores of the two primary outcomes, fatigue and physical functioning, at the end of treatment, can be seen in Table 1. Follow-up scores were not provided. Table 1 shows that these scores are such that participants would still be ill enough to be treated with GET, but also with CBT, in the aforementioned PACE trial [54], another study by White.

In addition to that, the GETSET trial itself noted that “we did not measure any objective outcomes, such as actigraphy, which might have tested the validity of our self-rated measures of physical activity” (p. 372 [57]). Consequently, the GETSET trial researchers were not sure if exercise therapy, based on the principles of GET, actually led to a real improvement for patients with PEM or not.

The German Institute for Quality and Efficiency in Healthcare (IQWiG) stated the following about the GETSET trial. “Overall, for the comparison of GET versus SMC for the endpoint physical performance for the short [12 weeks or end of treatment] and medium-term [52 weeks] evaluation period, there is no indication of an advantage or disadvantage of GET compared to the specialist medical care (SMC) control group” (in German: “Insgesamt ergibt sich für den Vergleich GET versus SMC für den Endpunkt körperliche Leistungsfähigkeit für den kurz- und mittelfristigen Auswertungszeitraum kein Anhaltspunkt für einen Vorteil oder Nachteil der GET im Vergleich zur SMC” (p. 131 [5]). Moreover, it also concluded that “there was a statistically significant difference in GETSET 15 months after randomization based on the adjusted mean difference in favour of the SMC control group” in regard to fatigue (in German: “in GETSET [ergab es] nach 15 Monaten nach Randomisierung auf Basis der adjustierten MWD [Mittelwertdifferenz] ein statistisch signifikanter Unterschied zugunsten der Kontrollgruppe SMC” zum Endpunkt Fatigue (p. 107 [5]).

#### 3.1.7. In Conclusion

White et al. finish this section by stating that “in summary, adopting PEM as a mandatory symptom for previous trial participants was not based on robust evidence. Therefore, downgrading the certainty of evidence on this basis (of indirectness or applicability) was inappropriate” (p. 3 [6]). Yet, they should have questioned the robustness of erasing PEM as a mandatory symptom of ME/CFS and replacing it with chronic fatigue when authors of White et al. were involved in creating the Oxford criteria in 1991. Just like they should have questioned the robustness of making PEM an optional criterion only when authors of White et al. were involved in creating the Fukuda (CDC) criteria in 1994.

### 3.2. Omission of Primary Outcome Data from Standard Trial End Points Used to Assess Efficacy

#### 3.2.1. End-Point Timings in a Fluctuating Disease

According to White et al., “the NICE committee did not use data from all time points and overlooked predefined end-point timings of trials” as “the NICE committee only considered outcomes for each trial at the data point furthest away from randomisation” (p. 3 [6]). White et al. state that because the primary endpoint in the PACE trial was 12 months and not 2 1/2 years after randomisation. They also state that it “was unsurprising that no significant differences in the primary outcomes (of fatigue and physical function) were observed…by this time” [long-term follow-up or 2 1/2 years after randomisation] because “44% of PACE trial participants had received either another course of the original therapy allocated or another trial therapy” (pp. 3–4 [6]). Moreover, according to them “any longer-term follow-up is purely naturalistic and outcomes, good or bad, are progressively less attributable to the original treatment to which the participants were randomised” (p. 3 [6]). Also, that “this naturalistic follow-up finding was used by NICE to conclude incorrectly that these treatments were essentially ineffective. The 6 and 12 months’ findings of clear benefit for both CBT and GET, from the largest clinical trial in the literature [the PACE trial], were not evaluated” (p. 4 [6]). However, there are a number of issues with these statements. For example, the supplementary appendix of the PACE trial’s long-term follow-up article, which formed part of the original submission and has been peer-reviewed [60], shows that 76%, 83% and 92% of the participants did not have any additional CBT, GET or APT (adaptive pacing therapy), respectively, after the trial had finished. Consequently, only 24%, 17% and 8% of participants had additional CBT, GET or APT, respectively after the final 52-week trial outcome assessment and before long-term follow-up.

Also, one of the authors of White et al. (Wessely) criticised NICE in the Stakeholder position statement on the NICE guideline for depression in adults by stating the following. “NICE should conduct a proper analysis of 1 and 2-year follow-up data from trials and *prioritise* [italic by us] treatment recommendations made on the basis of these data over and above recommendations which are made on the basis of short-term outcomes (less than 1 year)” (p. 5 [61]). The results at 52 weeks in the PACE trial are, in reality, measured six months after the end of treatment, which means that these results should not be prioritised because recommendations should not be made on the basis of short-term outcomes, when the follow-up is less than 1 year, according to the above-mentioned author of White et al. (Wessely) [6,61]. Moreover, an influential systematic review by Whiting et al. from 2001 about the interventions for the treatment and management of ME/CFS concluded that because ME/CFS is by definition a long-term condition with a relapsing nature, “that follow-up should continue for at least an additional 6 to 12 months *after the intervention period has ended* [italic by us], to confirm that any improvement observed was due to the intervention itself and not just to a naturally occurring fluctuation in the course of the illness” (p. 1367 [62]). This means that just like NICE, but also just like one of the aforementioned authors of White et al. (Wessely), they considered long-term follow-up data of treatments to be more reflective of efficacy in real life in a naturally fluctuating disease than short term follow-up data.

#### 3.2.2. Long-Term Follow-Up Results of the PACE Trial

As mentioned earlier, the three principal investigators of the PACE trial are all involved in White et al. and they have repeatedly claimed that long-term follow-up (LTFU) showed that CBT and GET are effective [63,64,65]. For example, in their long-term follow-up article, they state that “the main finding of this long-term follow-up study of the PACE trial participants is that the beneficial effects of the rehabilitative CBT and GET therapies on fatigue and physical functioning observed at the final 1 year outcome of the trial were maintained at long-term follow-up 2·5 years from randomisation” (p. [60]).

Also, in response to the re-analysis by Wilshire et al. of the PACE trial [41], Sharpe et al. state that “after carefully reviewing Wilshire et al.’s criticisms of the PACE trial findings, we can find no good reason to change its conclusions” and they continue to claim that “long-term naturalistic follow-up of trial participants…found that the benefits of CBT and GET were maintained 18 months after the end of the trial” (pp. 2,4 [66]). The three principal investigators of the PACE trial also state that in their response to the PACE-Gate editorial by Geraghty, and White does so again in a *HealthSense* newsletter in the spring of 2022 [67,68,69]. It also means that around six months after the updated NICE guideline was published in October 2021, White is still claiming that long-term follow-up shows that CBT and GET are effective. Yet, in his current anomaly article, which was submitted to the journal on the 25 September 2022 and accepted on the 3 May 2023 [6], he now disagrees with the fact that NICE uses long-term follow-up as the main outcome. Why he is now unhappy about using long-term follow-up in view of his previous claims of efficacy at long-term follow-up is unclear.

White et al. also state that “a cursory look at other current NICE guidelines to related or overlapping long-term conditions shows that this [using long-term follow-up data] is not standard practice for NICE” (p. 4 [6]). Yet, the reference at the end of the sentence refers to only one guideline (chronic pain) and other current guidelines are not used, despite “other current…guidelines” being plural. Moreover, the NICE guideline for chronic pain states the following about exercise. “Evidence from many studies showed that exercise reduced pain (23 studies) and improved quality of life (22 studies) compared with usual care in people with chronic primary pain. Benefit was seen for both short- *and long-term follow up* [italic by us] and was consistent across different types of exercise” (p. 24 [70]). In other words, this guideline did not ignore long-term follow-up data. This NICE guideline for chronic pain also looked at CBT. It concluded that “CBT for pain improved quality of life for people with chronic primary pain. A consistent benefit was not demonstrated in other outcomes”, but also that “the evidence was not of high quality so they [the committee] decided to recommend that CBT (for pain) is considered, rather than making a stronger recommendation to offer CBT (for pain)” (p. 25 [70]). So, this NICE guideline also noted problems with the quality of CBT studies. Moreover, this committee did not take into account the problem of using subjective outcomes in non-blinded trials with badly designed inactive control groups like usual care. Yet, according to psychology professor Cuijpers in an article entitled *How to prove that your therapy is effective, even when it is not?*, this is one of the methods that are available to help you show that your therapy is effective, even when it is not [71].

Looking at the results of the two primary outcomes of the PACE trial at 6 and 12 months, as requested by White et al., shows the following. The fatigue and physical functioning scores after treatment with CBT and GET are such that patients are still ill enough to re-enter the PACE trial to be treated with the same treatments, as can be seen in Table 2 and Table 3. According to White et al., NICE used the long-term follow-up outcomes of the PACE trial “to conclude incorrectly that these treatments were essentially ineffective” (p. 4 [6]). Yet, Table 2 and Table 3 show that the two primary outcomes at the end of treatment and six-month follow-up of the PACE trial itself confirm that conclusion by NICE.

### 3.3. Discounting Trial Data When Assessing Treatment Harm in Favour of Lower-Quality Reports

#### 3.3.1. Email to Investigate the Safety of CBT

White et al. state that “harm is a critical issue to consider for all treatments, including psychological and physical therapies… The NICE committee inverted the usual evidence hierarchy by not adequately considering the reassuring evidence of the low risk of treatment harms found within randomised controlled trials, of GET in particular. Instead they prioritised qualitative studies and patient organisation surveys” (p. 4 [6]). However, one of the authors of White et al. (Knoop) wrote an email on 4 August 2023 [73] to the patients who have been treated in his fatigue clinic for chronic fatigue and ME/CFS to investigate the safety of CBT, which contains an element of GET (graded activity), when used for ME/CFS. He asked for that information because of a new ME/CFS guideline in the Netherlands which should be ready in 2026. In the email, he writes: “Patient associations and the Patient Federation of the Netherlands have drawn up a questionnaire to map the experiences with ME/CFS patients. These experiences are important input in drawing up the new guideline for healthcare providers” (in Dutch: “Patiëntenverenigingen en de Patiëntenfederatie Nederland hebben een vragenlijst opgesteld om de ervaringen met ME/CVS patiënten in kaart te brengen. Deze ervaringen zijn belangrijke input bij het opstellen van de nieuwe richtlijn voor zorgverleners” (p. 1 [73]). Writing that email suggests that Knoop acknowledges the fact that randomised controlled trials might claim that CBT is safe, but they do not actually provide “reassuring evidence of the low risk of treatment harms”. Because if those studies would have provided that, then there would have been no need to write that email to investigate the safety of CBT. Moreover, according to a Dutch multidisciplinary ME/CFS guideline from 2013, which included one of the authors of White et al. (van der Meer), it is not unusual for interventions that are safe and effective in a research setting to perform less well in real life outside the confines of clinical trials [74]. Additionally, the post-market safety of 222 novel therapeutics after the initial regulatory approval was investigated by Downing et al. [75]. They found that there were safety issues with many (32.0%) novel therapeutics in general and with psychiatric therapeutics in particular. By definition, post-market safety issues arise long after clinical trials have finished. As a consequence of that, those safety issues are brought to the fore by patients and not by the researchers whose studies have long finished. Moreover, patients are the ones who are treated with a treatment and hence they are the ones who are at risk of being harmed by treatments, making it logical to rely heavily on their post-market account of the safety of a treatment.

#### 3.3.2. Meta-Analysis by White and Etherington

White et al. acknowledged that “the latest meta-analysis [from 2022 by Chou et al.] suggested that previous trials had limited reporting of harms”, but “NICE was … provided with a summary of a meta-analysis of harm data from all 10 published trials of GET. This meta-analysis found no excess evidence of harm in relation to either the number of participants withdrawing from GET or rating their overall health as worse after treatment, when compared with controls” (p. 4 [6]). However, they do not mention that this meta-analysis of harm data was carried out by White and Etherington [76], two of the authors of White et al. [6]. They also do not mention that Professor White himself was the principal investigator of three of the ten studies in that analysis. Moreover, they do not mention that not everybody who deteriorated was labelled as deteriorated by White and Etherington as the CGI [clinical global impression change scores of overall health] was used as part of the definition of safety. Yet, a “CGI score of 5 (“a little worse”) was not included in our measure of deterioration” (p. 3 [76]). White and Etherington also noted the following. “Sometimes trials had more than one possible control intervention, so a choice had to be made. For the FINE trial, the control intervention chosen was supportive listening, since by definition no one dropped out of GP care” (p. 3 [76]). Yet, according to the FINE trial itself, “our control condition is treatment as usual by the general practitioner” (p. 1 [77]) and not supportive listening as chosen by White and Etherington [76]. Consequently, not everybody who deteriorated after GET was counted as deteriorated in the meta-analysis by White and Etherington, and for the FINE trial, they used the wrong control group, which might have artificially inflated the results further.

Additionally, 8 of the 10 trials that were included in the meta-analysis by White and Etherington [76] were also included in the amended Cochrane exercise review by Larun et al. [78], which, according to PROSPERO, the International prospective register of systematic reviews, included two of the authors of White et al. (Flottorp and Glasziou) [79]. Larun et al. concluded that “the impact of exercise therapy on serious adverse reactions is uncertain” because most studies did not report about safety or “because the certainty of the evidence is very low” (p. 37 [78]).

All 10 studies that were included in White and Etherington were also included in a review by Chou et al. [80] for the American Centers for Disease Control and Prevention (CDC). Chou et al. concluded that harms of graded exercise or CBT versus inactive controls were not well reported, but also that there was “limited evidence that exercise and CBT were not associated with increased risk of serious adverse events or worsening of symptoms” (p. iii [80]). The aforementioned AHRQ evidence report also concluded that “harms are generally poorly reported” (p. 1 [37]). Finally, as noted by a systematic review of CBT and GET for ME/CFS by Ahmed et al., “not reporting adverse events is typical for this field as psychotherapy trials generally report infrequently on adverse outcomes” (p. 13 [35]).

#### 3.3.3. Consensus Statement on the Risks of Physical Activity in People Living with Chronic Diseases

In 2022, Reid et al. developed a consensus statement on the risks of physical activity for people living with long-term conditions on behalf of the British Physical Activity Risk Consensus group. They concluded that “a challenge for this study is that the risk of physical activity-related adverse events in people living with LTCs [long term conditions] is seldom reported and poorly quantified” (p. 434 [81]). They also noted that a limitation of their study was that their “list of medical conditions covered is not exhaustive. For instance, we do not include chronic fatigue syndrome and long COVID-19 *since evidence on physical activity risk is limited* [italic by us]…we cannot be sure that our symptom-specific statements translate effectively to clinical practice and subsequently to people living with these conditions” (p. 434 [81]).

Moreover, a systematic review by Casson et al. that was also used by White et al. in support of their claim that CBT and GET are effective, even though it was a systematic review of pacing, noted that graded exercise therapy might not be safe because “the addition of GET to a weekly routine could lead to increased risk of symptom exacerbation because of the combined effect of the GET with other incidental physical activities and cognitive activities” (p. 13 [82]).

#### 3.3.4. High Dropout Rate

Additionally, White et al. [6] did not mention the analysis of a 12-month program of GET in a sports medical department of a Dutch hospital and the very high dropout rate of 55%, 73%, and 80% at 6, 9, and 12 months, respectively, as found by a reanalysis [46]. Also, clients who remain in treatment tend to be higher functioning than those who drop out but they are unrepresentative of the clients who initially enrolled, as concluded by psychology professor Lilienfeld [83]. He also concluded that “clients who are not improving are especially likely to leave psychotherapy. As a result, therapists may conclude erroneously that their treatments are effective merely because their remaining clients are those that have improved” (p. 367 [83]). He also noted that “clients who dropped out may not have been helped or may have even been harmed by the intervention” (p. 367 [83]).

#### 3.3.5. The Levels of Evidence and Patient Surveys

As noted by Burns et al. in an article entitled *The Levels of Evidence and their role in Evidence-Based Medicine*, “although RCTs [randomised controlled trials] are often assigned the highest level of evidence, not all RCTs are conducted properly and the results should be carefully scrutinized” (p. 2 [84]). NICE concluded that CBT and GET studies were all of low or very low quality. Qualitative studies and patient organisation surveys are then not only the next level of evidence but also become more important to assess the safety of CBT and GET because of the lack of quality of the RCTs. Kindlon [85] who pooled patients’ surveys, noted that the first survey that documented problems with the safety of CBT and GET was the ME Action survey from 1990. This charity became later known as Action for ME. The surveys of this charity are of particular interest because Dr Miller, one of the authors of White et al., was Principal Medical Adviser for Action for ME from 2010 until 2016 [86] and according to a survey from that period, GET was harmful in 60% of cases [87], as can be seen in Table 4. Other surveys from the same charity also highlight the harmfulness of CBT and especially GET.

White et al. also do not mention the study by the Oxford Brookes University [89] (*n* = 2274) from 2019 on the safety of CBT and GET, which was commissioned by NICE as part of their review of the ME/CFS guidelines. Worsening of symptoms after treatment was reported by 58.3% (CBT) and by 81.1% (GET). In addition, 14% (CBT) and 22.4% (GET) ended up bedridden and dependent on help from others due to severe ME/CFS. Finally, a survey by the 25% ME group, the charity that represents the people who are bedridden with severe ME, concluded in 2004 (*n* = 437) that 82% of them had been made worse by GET [85].

#### 3.3.6. The MAGENTA Trial

Also, the MAGENTA trial (*n* = 241) by Gaunt et al. [90] compared GET (123 participants) with activity management (AM, 118 participants) in children aged 8–17 years. The primary outcome was physical function after 6 months. Gaunt et al. concluded that “there was no evidence that GET was more effective or cost-effective than AM … by the 6-month or 12-month assessment points” (p. 1 [90]). The accelerometer data showed that “the MAGENTA participants had a reduction in moderate-to-vigorous-intensity physical activity at 3 and 6 months” (p. 7 [90]). Consequently, the accelerometer data show that GET had a negative effect on the health of participants and therefore contradicts the claim by White et al. that GET is safe.

#### 3.3.7. In Conclusion

Finally, White et al. finish this section by concluding that “while systematic studies of the safety of GET found no convincing evidence of harm with GET, NICE concluded that GET was not safe” (p. 4 [6]). Yet, as noted earlier by, for example, the Cochrane exercise review, most studies did not report about safety, but even if they would have, safety issues often arise when treatments are used in real life, outside of the realms of RCTs and long after those have finished. Moreover, RCTs of CBT and GET are of low or very low quality and suffer from many methodological and other limitations, including cherry-picking its participants. The PACE trial [54], for example, screened 3158 patients for eligibility, yet only 641 (20.3%) were selected, which suggests that cherry-picking was a problem of that study. According to White, the main principal investigator of the PACE trial, their study relied on subjective outcomes because “who better to know whether these treatments work than patients themselves?” (p. 6 [69]). Why White et al. then ignore that patients have been saying for decades that CBT and GET are ineffective and harmful, and why they do not want NICE to use surveys from patient organisations, if patients are the ones who know best if treatments work or not, is unclear.

### 3.4. Minimisation of the Importance of Fatigue as an Outcome

#### 3.4.1. Subjective Outcomes

White et al. state that “the NICE committee decided to downgrade all fatigue outcomes based on the premise that it is a subjective measure. This was inconsistent with the diagnosis of CFS/ME; all definitions depend on self- reported symptoms that are by definition, subjective” (p. 4 [6]).

Why the authors state the opposite to what NICE concluded is unclear as NICE emphasised the importance of fatigue as an outcome. In a section entitled “The outcomes that matter most”, NICE stated that “*fatigue*/fatigability [italic by us], physical function, cognitive function, psychological status, pain, sleep quality, treatment-related adverse events, activity levels, return to school/work and exercise performance measures were considered by the committee to be *critical outcomes for decision making*” [italic by us] (p. 64 [1]). Moreover, it also stated that “*fatigue*/fatigability [italic by us], unrefreshing sleep and physical and cognitive dysfunction are recognised as *key symptoms of ME/CFS* [italic by us]. The worsening or improvement of these symptoms reflect the impact of an intervention or strategy” (p. 64 [1]). Key symptoms and critical outcomes for decision-making do not sound like downgrading fatigue based on the premise that it is a subjective measure. As a matter of fact, 7 of the 10 critical outcomes for decision-making are subjective measures. So, rather than downplaying fatigue’s importance as an outcome, NICE highlighted it as a key symptom of ME/CFS. Indeed, according to NICE, fatigue is one of the “outcomes that matter most” (p. 64 [1]).

#### 3.4.2. Non-Blinding and Exaggeration of Intervention Effects

White et al. also state that “the NICE committee took the view that therapy trials not being ‘blinded’, with both participant and therapist being aware of the intervention, impaired the validity of the results. Trials of complex non-pharmacological interventions often necessitate non-blinding of participants and therapists. Interestingly, a recent meta-epidemiological study of 142 Cochrane trial meta-analyses concluded that concerns over bias by lack of blinding in randomised trials may have been exaggerated” (p. 4 [6]). However, that meta-epidemiological study by Moustgaard et al. [91] does not say that those concerns are incorrect. It only notes that those concerns about lack of blinding may have been exaggerated. It also notes that “non-blinded outcome assessors of subjective outcomes exaggerated odds ratios by 36%, on average” and that “a systematic review by Page and colleagues found…an exaggeration of 23% when outcomes were subjective” (p. 11 [91]). In other words, that recent meta-epidemiological study of 142 Cochrane trial meta-analyses confirms that the combination of lack of blinding with subjective outcomes in randomised trials leads to an overestimation of the treatment effect, which makes subjective outcomes in non-blinded studies unreliable. A similar exaggeration of intervention effects was found by the BRANDO project (Bias in Randomised and Observational studies) [Savović], which, amongst others, included Stanford Professor Ioannidis. The BRANDO project concluded that as a consequence, clinical and policy decisions should not be based on trials in which blinding is not feasible and outcome measures are subjectively assessed. They finished by stating that “therefore, trials in which blinding is not feasible should focus as far as possible on objectively measured outcomes” (p. 45 [92]).

More importantly, though, is how reliable subjective outcomes in non-blinded trials for ME/CFS are. White et al. do not ask or answer that question but it was answered by the reanalysis of the amended Cochrane exercise review in 2020 [93]. Their analysis of the objective outcomes from three CBT and GET trials for ME/CFS confirmed the unreliability of subjective outcomes in non-blinded studies for ME/CFS, as shown by the following examples:

1. There was a substantial difference in subjective physical functioning scores at baseline between the exercise and control group in Jason et al. [94], yet objectively there was not (6 min walk test or 6MWT);
2. Physical functioning subjectively improved by 30% after GET in Moss-Morris et al. [95], yet objectively it deteriorated by 15% (CPET);
3. The released individual participant data from the PACE trial by White et al. showed that 20% of participants who had improved subjectively (physical functioning) had deteriorated objectively (6MWT) [54,93,96,97].

Moreover, as noted by Monaghan et al. [98] in an article entitled *Blinding in Clinical Trials: Seeing the Big Picture*, “we blind because the potential for bias is everywhere. Bias can take numerous shapes and forms when people involved in a research study are privy to information about the assigned interventions. Participant knowledge of their group allocation can bias expectations, adherence to the trial protocol, treatment-seeking behaviour outside the trial, and assessment of the effectiveness of an intervention. Differential treatment, attention, or attitudes toward subjects by a non-blinded healthcare team or other members of the research staff also pose a major threat to unbiased outcomes. Importantly, once bias is introduced from any one of these potential sources, there exist no analytical techniques by which to reliably correct for this limitation” (p. 2 [98]).

#### 3.4.3. The Primary Intention of CBT for ME/CFS

White et al. also state that NICE provided the wrong description of what CBT entails by suggesting “that CBT should focus primarily on support for managing symptoms and treating (emotional) distress, which was seen as a consequence of the illness. This is not what CBT was developed to do or how it was delivered in the trials for CFS/ME. The primary intention of CBT in the context of CFS/ME is to improve fatigue and function” (p. 4 [6]). However, what NICE concluded is that CBT, as developed for ME/CFS, and thus with graded activity, does not lead to improvement or recovery. Instead, CBT, as developed by Beck in the 1960s [99], should be offered to patients if they suffer from depression or anxiety or need help coping with their disease. A number of authors of White et al. (Chalder, David, Sharpe and Wessely) [12,13] modified CBT as developed by Beck [99], based on their CBmodel, to make it a treatment for ME/CFS. Yet, they did not change its name and, by doing so, they created a Babylonian confusion of tongues, and now they complain about that.

#### 3.4.4. Meta-Analyses and Systematic Reviews

White et al. continue to claim that “a treatment such as CBT that reduces fatigue and improves functioning is therefore a treatment that improves the condition, as the clinical trial evidence shows” (p. 4 [6]) by referring to a number of meta-analyses and systematic reviews, for example, the Cochrane CBT review by Price et al. from 2008 and the amended Cochrane exercise review by Larun et al. from 2019 [78,100]. White et al., however, do not mention that The Cochrane Library has put an editorial note on the CBT review by Price et al., stating that “the review is no longer current. It should not be used for clinical decision-making” [101]. White et al. mention the amended Cochrane exercise review, yet they do not mention the reanalysis of it which found that there were many problems with the review and the studies in it, including not taking into account that GET had a negative instead of a positive effect on work status [93], which was also found by a reanalysis of the original Cochrane exercise review as well as by an extensive review of the literature into the effect of CBT and GET on work and disability status [42,102].

White et al. also use the systematic review by Ingman et al. from 2022. One of its authors (Chalder) is also an author of White et al. [6]. According to White et al., that systematic review concluded that “results suggest some support for the positive effects of CBT and GET at short-term to medium-term follow-up” (p. 2 [6]). Yet, Ingman et al. also noted that “however, previous findings concerning short-term to medium-term follow-up are inconsistent” (p. 11 [103]). Consequently, Professor Chalder concluded herself that there is not a lot of evidence that both treatments are effective. This is even more interesting because 3 of the 15 studies in that review were conducted by herself, including the PACE trial [54], of which she was one of the three principal investigators, and the two other principal investigators (White and Sharpe) are also part of White et al. [6]. In addition, White et al., with Chalder, argue that it was wrong for NICE to look at outcomes at long-term follow-up and that NICE should have looked at short-term to medium-term follow-up, which, according to them, shows that CBT and GET are effective [6]. Yet, that is contradicted by Chalder when she was an author of the systematic review by Ingman et al. [103].

Also, Ingman et al. concluded that one of the limitations was “that most studies reported subjective, self-report measures, which may have increased the risk of observer or detection bias” and that “subjective and objective measures do not necessarily correlate” (p. 11 [103]). They therefore concluded that “future trials should report objective outcomes, in addition to self-report measures” (p. 10–11 [103]). Consequently, Ingman et al., with Chalder, contradict the aforementioned statement by White et al., also with Chalder, “that concerns over bias by lack of blinding in randomised trials [by relying on subjective outcome measures] may have been exaggerated” (p. 4 [6]).

#### 3.4.5. Objective Outcomes

It is unclear why the review by Ingman et al., just like the meta-analyses and other reviews used by White et al. to claim that CBT and GET are effective, ignored the objective outcomes. Even more so because an evaluation study from 2021 into the efficacy of GET in a specialist clinic in South London, which also included Chalder, concluded that “our study utilised self-report measures which can be problematic because they are dependent on a patient’s perception of their own illness” (p. 7 [104]). A retrospective longitudinal study by Stevelink et al., which was also from 2021, and which also included Chalder, concluded that “given the evidence that meaningful occupation is important for well-being and psychosocial needs, work-related outcomes should be targeted in CFS treatment” (p. 6 [105]). A mini-review of the literature concluded that “if the PACE trial’s reported results of significant improvement and recovery were accurate than a measurable benefit in employment and in the receipt of financial support would have been expected” (p. 1237 [106]). Yet, “the objective data and outcomes [of the PACE trial] indicate that CBT and GET do not lead to regularly apparent benefits in this domain” (p. 1238 [106]). Why Stevelink et al. and White et al. ignore these results from the PACE trial is unclear. It is also unclear why they ignore an extensive review of the literature [42] which found that CBT and GET do not restore the ability to work, and have a negative effect on work status and illness/disability benefit status and not a positive.

The systematic review by Ahmed et al. [35], which is not mentioned by White et al., concluded that “in order to securely demonstrate the efficacy of CBT/GET within a non-blinded design, researchers need to show that self-reported improvements are supported by objectively measurable outcomes and report on the harms/adverse events” (p. 13 [35]). As can be seen in Table 5, the objective outcome measures of CBT and GET studies show that these treatments do not lead to objective improvement, highlighting the inefficacy of both treatments. Consequently, the objective outcome measures of these studies confirm what patients have been saying about the inefficiency of these treatments since the early 1990s.

The German IQWiG Institute stated the following about the six-minute walk test (6MWT) in the PACE trial. “Given the mild to moderate disease severity of the included study population, the difference of at least 8.17 m [end of treatment (after 24 weeks)] or 16.70 m [six months follow-up (after 52 weeks)] (lower Cl limits of the estimates) between the treatment groups appears to be too small” (in German: “Angesichts der leichten bis moderaten Krankheitsschwere der eingeschlossenen Studienpopulation, erscheint der Unterschied von sicher 8.17 m [nach 24 Wochen] bzw. 16.70 m [nach 52 Wochen] (untere Kl-Grenzen der Schätzungen) zwischen den Behandlungsgruppen zu gering” (p. 131 [5]).

Diseases like pulmonary hypertension or lung emphysema differ too much from ME/CFS to use their Minimal Clinical Important Difference (MCID) on the 6MWT to compare it with the aforementioned change of 8 and 16 m, as was carried out by IQWiG [5]. A study to determine the MCID on the 6MWT in ME/CFS has not been carried out. However, according to a systematic review by Silva-Passadouro et al., Fibromyalgia, ME/CFS and Long COVID are similar multisymptom clinical syndromes [120]. Buchwald, one of the authors of White et al. [6], concluded that “despite remarkably different diagnostic criteria, CFS and FM [fibromyalgia] have many demographic and clinical similarities. More specifically, few differences exist in the domains of symptoms, examination findings, laboratory tests, functional status, psychosocial features, and psychiatric disorders” (p. 236 [121]). Santhouse, another author of White et al. [6], concluded that “there is considerable overlap between the symptoms of fibromyalgia and CFS” (p. 648 [122]). Finally, Wessely and Sharpe, two of the worldwide leading proponents of CBT and GET for ME/CFS, who are also authors of White et al. [6], concluded not only that the symptomatology of fibromyalgia overlaps with CFS. But also that it is essentially the same condition [123]. Finally, White et al. noted that “fibromyalgia…overlaps substantially with CFS/ME in terms of comorbidity and current aetiological and mechanistic thinking” (p. 5 [6]). We mention this because a study to determine the MCID on the 6MWT in fibromyalgia was carried out by Kaleth et al. (*n* = 187) [124]. They concluded that “the MCID for 6MWD [six-minute walk distance] in patients with FM was 156 to 167 m”. But also that “these findings provide the first evidence of the change in 6MWD that is perceived by patients to be clinically meaningful” (p. 1 [124]). The above-mentioned changes of 8 m, respectively 16 m after GET in the PACE trial, were trivial compared to the MCID of 156 to 167 m. Moreover, fitness did not improve in the PACE trial. This makes it likely that these minimal changes were simply artifacts caused by all the methodological problems, biases and confounding factors of the study, instead of a real improvement. Moreover, it is also well within the 10% of the learning effect on this outcome measure which is achieved by simply repeating the test yet this does not represent a real improvement brought about by the treatment either [125].

The aforementioned AHRQ evidence report concluded that there is “insufficient evidence of the effectiveness of GET on any outcome” (p. 13 [37]) after excluding the trials using the Oxford case definition for inclusion. This is in line with the conclusion from the German IQWiG Institute, which did not exclude those studies, that “overall, for the comparison of GET versus SMC for the endpoint physical performance for the short and medium-term evaluation period, there is no indication of an advantage or disadvantage of GET compared to SMC” (in German: “Insgesamt ergibt sich für den Vergleich GET versus SMC für den Endpunkt körperliche Leistungsfähigkeit für den kurz- und mittelfristigen Auswertungszeitraum kein Anhaltspunkt für einen Vorteil oder Nachteil der GET im Vergleich zur SMC” (p. 131 [5]).

#### 3.4.6. Issues with the Chalder Fatigue Scale

White et al. emphasise the importance of fatigue as an outcome, yet they ignore that there are many issues with the frequently used Chalder fatigue scale (ChFS). Studies that use this questionnaire are, for example, the PACE trial, and its sister trial, the FINE trial [54,113]. The reanalyses of the Cochrane GET and CBT reviews listed a total of 10 different problems with it [102,125]. Some of the most important ones are:

- The ChFS does not provide a comprehensive reflection of fatigue related severity, symptomology or functional disability in ME/CFS;
- The ceiling effect is an important issue;
- Most items on the ChFS do not clearly relate to fatigue;
- The ChFS is not able to distinguish between primary depression and ME/CFS;
- The ChFS has limited evidence of test–retest reliability.

The above-mentioned ceiling effect is an important problem of the ChFS. This means that a maximum score at baseline cannot increase, even if there is deterioration during the trial. Analysis of the released individual participant data of the PACE trial and the FINE trial [96,97,102,126] showed that a large percentage of participants in both trials had maximum scores at baseline. A total of 75% (FINE) and 65% (PACE) had the maximum bimodal score (scoring: 0, 0, 1, 1; 0–11) and 29% (FINE) and 15% (PACE) had the maximum Likert score (scoring: 0, 1, 2, 3; 0–33). If patients with maximum Likert scores at baseline, for example, had improved on three of the eleven items, and deteriorated on eight, then they should have been classified as deteriorated by five points (eight minus three). But as the eight scores could not get worse, then these patients would be classified as having improved by three points. An outcome measure that labels patients who have deteriorated as improved is not a very accurate and reliable instrument. Moreover, ceiling effects are classified depending on the percentage of participants scoring the highest possible score on that measure [127]. They are classified as:

- Significant if ≥15% do so;
- Moderate if 10% to <15% do so;
- Minor if 5% to <10% do so;
- Negligible if <5% do so.

Consequently, the ceiling effect is a significant problem of the Chalder fatigue scale in the PACE trial and the FINE trial and most likely in many other ME/CFS trials. Patient-reported outcome measurements that exhibit a significant ceiling effect, as illustrated by the aforementioned percentages in the PACE trial and the FINE trial, show clustering of participants’ scores towards the upper limit of a scale and consequently have low discriminatory power among high-end scores. As a result, the ability of the ChFS to detect changes over time and to discriminate differences between groups is very low. It also suggests limited instrument range, measurement inaccuracy, and response bias [127,128], all of which indicate the inaccuracy of an assessment tool. The ChFS is therefore an unreliable instrument and is unsuited to be used in ME/CFS.

The unreliability of this instrument was highlighted by a study by Tench et al. [129] on the efficacy of GET for SLE, which included one of the authors of White et al. (White) as found by the reanalysis of the amended Cochrane exercise review [93]. Tench et al. compared the efficacy of 12 weeks of GET with two other interventions (relaxation and no intervention) and used a number of subjective outcomes as well as objective ones (including VO2 peak). This study used three different measures for fatigue: the fatigue severity score, the ChFS and the visual analogue scale. The Chalder fatigue scores after GET improved by 3 points compared to relaxation and by 4 compared to the no-treatment control group [129]. This is similar to the improvement in the PACE trial [54]. If Tench et al. had been used by White et al., then they would have stated that this study provided evidence of the efficacy of GET for SLE at the end of treatment. However, GET did not lead to improvement on the other two fatigue scales, nor did it lead to objective improvement. Also, improvements in Chalder fatigue scores were not maintained at (three months) follow-up [93,129].

White et al. do not mention the systematic review by Ahmed et al. from 2019 [35]. The reviewers concluded that “the findings of this systematic review do not support the claim that CBT and GET are effective treatments for ME/CFS patients, due to methodological flaws and biases found in the studies that are investigated in this” (p. 13 [35]). One of the included studies was the PACE trial, which White et al. [6] use as their main evidence for their claim of efficacy of CBT and GET. According to Ahmed et al., the PACE trial suffered from performance and detection bias, reporting bias (selective reporting), and other forms of bias (p. 5 [35]). Ahmed et al. also concluded that “Prins et al. (2001) [the biggest CBT trial for ME/CFS from the Netherlands] and White et al. (2011) [the PACE trial; and two other studies] scored a high risk of bias in three or four categories, making them the [four] most biased studies” (p. 4 [35]).

In conclusion, one cannot safely conclude that CBT and GET are effective if patients have not improved objectively, and, after treatment, more patients are unable to work and reliant on illness and disability benefits than before treatment.

### 3.5. Non-Standard Use of GRADE (Grading of Recommendations, Assessment, Development and Evaluations) to Assess the Trial Evidence

#### 3.5.1. The GRADE Evidence to Decision Framework

White et al. state that “based on the published reviews of the trial evidence, we consider that the research evidence was not presented adequately by NICE, resulting in the decision-making process being less robust. The application of the GRADE Evidence to Decision framework fell short of international expectations” (p. 4 [6]). Yet, the reference at the end of this statement does not refer to the GRADE Evidence to Decision framework. Instead, it refers to an opinion article from 2021 by Turner-Stokes and Wade [130] and one of them (Wade) is also an author of White et al. [6]. Turner-Stokes and Wade write that “within the hierarchy [of research evidence], evidence from randomised trials starts high but is downgraded if trials fail to meet strict quality criteria”. They also state that “quality criteria…are poorly applicable to complex conditions” like ME/CFS (p. 1 [130]). Just like White et al., they are trying to argue that for behavioural interventions like CBT and GET, scrutiny should be less strict than for pharmaceutical interventions. But that implies that we should accept low-quality studies and low-quality evidence, whereas there is a simple measure (objective outcomes) to increase the quality of those studies. Moreover, the GRADE handbook itself notes that there are a number of study limitations in randomised controlled trials [131]: among them, a lack of allocation concealment, lack of blinding, and incomplete or absent reporting of some outcomes and not others on the basis of the results, which is known as selective reporting [131]. Also, Wade himself noted the following in a statement released on his behalf by the Science Media Centre, a communications agency with close ties to White et al.: “Randomised controlled trials provide the best and only reliable evidence on safety and effectiveness of any intervention in any condition” [132]. Only reliable evidence in any condition also means in complex conditions like ME/CFS.

Additionally, as concluded by Edwards in one of the articles in the Special Issue of the *Journal of Health Psychology* on the PACE trial [133], using subjective outcome measures in non-blinded studies is a key design flaw “demonstrating a lack of understanding of basic trial design requirements. The failure of the academic community to recognise the weakness of trials of this type suggests that a major overhaul of quality control is needed” (p. 1155 [134]). Consequently, if non-blinded studies rely on subjective outcomes, then the criteria should not be less strict; instead, they should be stricter because of all the different forms of bias caused by the combination of those two.

White et al. also state that “the original GRADES methodologists described the review as ‘… a disastrous misapplication of GRADE methodology, …’” (p. 4 [6]) and they refer again to the aforementioned article by Turner-Stokes and Wade from 2021 [130]. The Grading of Recommendations Assessment, Development and Evaluation (GRADE) Working Group presented its initial proposal for patient management in 2004. Turner-Stokes and Wade are not listed as members of the GRADE Working group [135], nor do Turner-Stokes and Wade write “a disastrous misapplication of GRADE methodology” (p. 4 [6]) in their criticism [130]. Why this is unknown to White et al. is unclear, because Wade is one of their authors, as noted above [6].

#### 3.5.2. Four Royal Colleges of Medicine

White et al. also state that four Royal Colleges of Medicine remarked about the “lack of a robust process of evidence synthesis and guideline development” by stating that “there is considerable disquiet in the medical profession and some patient groups about the way the data and evidence have been assessed” (p. 4 [6]) The wording, some patient groups, suggests that not one but more than one patient group are not happy about the guideline. Yet, we are not aware of any patient group, let alone more than one patient group, which is unhappy about that, and the authors do not make it clear to which groups they refer. The Colleges state that “Graded Exercise Therapy as defined in the guidance is not reflective of the personalised paced exercise programmes that are currently used in the NHS and termed GET. These have provided benefit to many patients and should not be discontinued. However, we recognise that the phrase GET is unhelpful and this terminology should be dropped to allow clinicians to work with their patients in a more productive way” (p. 1 [136]). What they are saying, however, is that they are not treating patients with GET anymore. The only reason why Royal Colleges would stop using a therapy is because it is not safe and effective. If it would have been, then they would not have stopped using it. Consequently, they acknowledge in an indirect way that the conclusion by NICE about GET is right. Moreover, it also means that they support the recommendation by NICE for pacing, which is opposed by White et al., too. Finally, it also means that the Royal Colleges use an energy management approach in the absence of supportive research evidence and, as we will see later, White et al. argue against such an approach by NICE. Yet, when the Colleges use it, they support this.

#### 3.5.3. Grading by an Independent NICE Technical Team

Psychologist professor Hughes noted about the process of evidence synthesis and guidelines of development that “it is important to note that the grading was carried out by an independent NICE technical team, who do this job for all the NICE reviews. In other words, it was not conducted by the ME/CFS review group. This reflects a standard approach applied by NICE to the evaluation of evidence bases for medical treatments” (p. 1 [137]). Moreover, the independent NICE technical team based their grading on the NICE guidelines manual (PMG6), process and methods from 2012 [138].

Finally, as can be seen in Table 5, the studies that used objective outcome measures ignored the fact that these outcome measures showed that their conclusion, that their treatments were effective, was incorrect. Put differently, all these studies resorted to selective reporting, which is not how researchers should assess and present trial evidence. Consequently, it was not NICE, but a number of authors of White et al. who resorted to a non-standard and incorrect way to assess and present their trial evidence to avoid publishing a null effect.

### 3.6. Interpretation of GET as Employing Fixed Increments of Change When the Major Trials Defined It as Collaborative, Negotiated and Symptom Dependent

#### 3.6.1. The CMO’s Working Group Report from 2002

According to White et al., NICE has wrongly defined GET by claiming that it is “incorporating fixed increments of exercise that are pursued irrespective of how the patient feels” (p. 4 [6]). According to them, “in GET, activity is determined collaboratively with the patient and only increased as the patient feels able, dependent on their symptomatic response” (p. 4 [6]). And they conclude by stating that “there are no ‘fixed increments of exercise’ in GET” (p. 5 [6]).

Three of the authors of White et al. (Chalder, Wessely and White) were involved in the writing of the Chief Medical Officer’s (CMO) Working Group report in 2002 [139]. The CMO report concluded that “graded exercise is a form of structured and supervised activity management that aims for *gradual but progressive increases* [italic by us] in aerobic activities such as walking or swimming. It is based on a principle—contested by some—that a principal factor maintaining the illness is inactivity, subsequent physical deconditioning, and its physiological consequences, which graded and supervised increases in exercise can help to reverse. In addition, it may act as a rehabilitative behavioural therapy by gradually exposing the patient to an activity (exercise) that has been avoided” (p. 46 [139]). The CMO report also noted that “one key controversy that exists over graded exercise rests on whether the nature of the treatment is appropriate for the nature of the disease, at least in some individuals”. They added that some think “that patients have a primary disease process that is not responsive to or could progress with graded exercise, and that some individuals are already functioning at or very near maximum levels of activity” (p. 46 [139]).

#### 3.6.2. The PACE Trial’s GET Manual for Therapists

White et al. also state that “we have been unable to find any trials that prescribed fixed increments of exercise” (p. 4 [6]). The GET manual for therapists (final version 7) of the PACE trial was written by three authors on behalf of the PACE trial management group [140], and one of them (White) is also the leading author of White et al. [6]. According to this manual, “the essence of GET is to help the participant to gradually engage and participate in physical activity and aerobic exercise” (p. 20 [140]). However, the PACE trial’s GET manual for therapists also makes it clear that “it is their *planned physical activity, and not their symptoms* [italic by us], that determine what they [participants] are asked to do” (p. 20 [140]). To make sure that the therapists understand the implication of this, this is repeated in the same manual when the authors state “planned physical activity and not symptoms are used to determine what the participant does” (p. 21 [140]).

The same manual also states about GET that “in contrast to APT [Adaptive Pacing Therapy], it is important that the “envelope theory” of pacing is not adhered to” (p. 69 [140]). However, also that “the rationale behind APT involves the ability of the body to heal itself by not provoking symptoms. In significant contrast, GET encourages the participant to stretch the limits of physical capacity in order to improve them” (p. 69 [140]). Yet, if patients stretch the limits of their physical capacity, then that leads to PEM, flare-ups and relapses. To make sure that the therapists understand the difference, the manual then states that “a main difference between APT and GET is that GET plans for *incremental increases in activity* [italic by us] while APT does not” (p. 69 [140]).

#### 3.6.3. Other Manuals

That exercise is not symptom-dependent also becomes clear from the following examples of statements from the CBT Manual for participants from the PACE trial [141], which was written by Burgess and Chalder. CBT for ME/CFS contains an element of GET (graded activity). Professor Chalder is one of the three principal investigators from the PACE trial and one of the authors of White et al. [6].

- “Most people experience one or two minor set-backs (increased symptoms) during treatment for one reason or another. [Yet] It is important to maintain the [activity] programme” (p. 19 [141]).
- “Your symptoms may slightly increase when you start your programme. However, this is usually only temporary and occurs as a result of changing your “usual” routine. Even though you may feel like resting more, it is important that you keep going with your activity programme. You will hopefully find that your symptoms will gradually decrease although this may take a few weeks” (p. 44 [141]).
- “You will be aiming to do things at regular times, *irrespective* [italic by us] of how you are feeling” (p. 18 [141]).
- “Persevere with your programme however difficult it may seem, and in time you will appreciate the benefits of gradually changing the way you do things” (p. 19 [141]).
- “**It is not** [sic] **necessary for your fatigue to have decreased for you to increase or start a new activity**” [sic] (p. 46 [141]).

The CBT Therapists manual from the same PACE trial also describes GET in a similar manner. “It is their *planned physical activity, and not their symptoms* [italic by us] that determines what they are asked to do” (p. 14 [142]). Moreover, one of the questions of the Graded Exercise Therapy Scale according to the GET manual for therapists from the PACE trial is, “did the therapist emphasis [sic] the importance of *incremental, progressive changes* [italic by us] in physical activity or exercise?” (p. 172 [140]). This is also stated about GET on page 119 in the CBT Therapists manual [142]. Consequently, the PACE trial prescribed fixed increments of exercise and not symptom dependent treatment.

White wrote the GET manual and one of the comments in the acknowledgement section of that manual is the following. “The treatment leaders and principal investigators have also made significant contributions towards defining GET and have contributed their time in making valuable comments” [to this GET manual] (p. 2 [140]). Why White et al. are not aware of all that is unclear because, as noted before, the three principal investigators of the PACE trial, who were also centre leaders (Chalder, Sharpe and White), as well as four other centre leaders (Angus, Murphy, Wade and Wessely) and one doctor (Miller) who was one of the three independent assessors of the trial safety data [54], are all authors of White et al. too [6].

#### 3.6.4. The GET Trial by Moss-Morris et al.

In the GET trial by Moss-Morris et al., “the importance of consistently meeting but not exceeding exercise targets was emphasized so that *the amount of exercise* undertaken each day *was not dependent on symptoms*” [italic by us] (p. 249 [95]). So, again, just like in the PACE trial, the amount of exercise participants have to undertake is fixed and not flexible or symptom-dependent.

#### 3.6.5. CBT’s Fixed Incremental Increases

It is not only GET which incorporates fixed incremental increases. According to the same GET manual from the PACE trial, this also applies to CBT, as highlighted by the following quote: “it [CBT] involves elements of simple pacing to stabilise activity, graded increases in activity, as with simple incremental pacing, and also directly addresses the participant’s beliefs and fears about their symptoms and functioning” (p. 18 [140]). The following wording in the same manual makes that even clearer. CBT contains “*planned increases* [italic by us] in activity with challenging of understanding of symptoms” (p. 22 [140]).

The CBT Therapists manual from the PACE trial also states about CBT that it is about “*planned increases in activity* [italic by us] with challenging of understanding of symptoms” (p. 16 [142]). Also, Chalder was one of the two authors of the CBT manual, and the two other principal investigators of the PACE trial (Sharpe and White) are thanked in the acknowledgement section of the CBT therapists manual, “for their comments and contributions” [to this CBT manual] (p. 2 [142]). Consequently, CBT is also about fixed increases instead of being symptom-dependent.

#### 3.6.6. In Conclusion

This all confirms the conclusion by NICE that fixed incremental changes are an important and fundamental part of GET. It also shows that these are an important and fundamental part of CBT for ME/CFS too. Consequently, NICE should also have concluded that CBT, as used for ME/CFS, is harmful too and should therefore not be used.

### 3.7. Inconsistency with NICE Recommendations of Rehabilitation Therapies for Related Conditions, Such as Chronic Primary Pain

According to White et al., it is “inconsistent” and “confusing for the outside world” that “NICE recommends both CBT and exercise therapies in a range of neurological conditions, such as multiple sclerosis, where it can reduce fatigue and improve mobility” but not in ME/CFS (p. 5 [6]). The reference at the end of that sentence refers to the NICE multiple sclerosis (MS) guideline in adults from 2014. However, that guideline was replaced by a new guideline in June 2022 [143]. The article by White was published a year later, in June 2023. Why they then refer to the old guideline is unclear. In the new MS guideline, NICE specifically states that “there was some evidence that fatigue or energy management interventions and wellbeing techniques, such as cognitive behavioural therapy (CBT) and mindfulness, are beneficial. However, the committee agreed that the evidence was not sufficient to recommend formal programmes because of limitations in the studies” (p. 41 [143]). Consequently, it is inconsistent and confusing for the outside world to claim that the NICE guideline for multiple sclerosis recommends CBT and exercise therapies when, in reality, it does not.

White et al. also claim that it is inconsistent to recommend CBT and exercise therapies for pain in one condition but not in another, i.e., ME/CFS [6]. Yet, for example, patients with heart failure can suffer from shortness of breath, just like patients with pneumonia. Antibiotics are recommended for the treatment of pneumonia, yet they are not recommended to treat shortness of breath in heart failure. Also, why should recommendations for different conditions be the same? And why would it be confusing for the outside world? Does relatedness between two conditions, by definition, not mean that those two conditions are separate and not the same condition? Consequently, it would be confusing for the outside world if there would not be a difference in recommended therapies. If everything would be the same, then there would also be no need to have an institution like NICE, whose job it is to create different guidelines for different conditions.

### 3.8. Recommendation of an Energy Management Approach in the Absence of Supportive Research Evidence

According to White et al., “NICE recommended ‘energy management’, in which patients are encouraged to stay within the energy limits imposed by their illness, and thus avoid exacerbating symptoms” which is known as pacing. Yet, according to them, “the only substantial evaluation of pacing for CFS/ME published to date was… the PACE trial.” And “the (limited) research evidence suggests” that it is “clearly less effective than either CBT or GET” (p. 5 [6]). However, the NICE guideline clearly states that energy management (pacing) “is a self-management strategy led by the person themselves” (p. 30 [1]), but also that it is “one of the most important tools that people with ME/CFS have to support them in living with the symptoms of ME/CFS” (p. 76 [1]). Moreover, NICE makes it very clear that it only “helps people learn to use the amount of energy they have while reducing their risk of post-exertional malaise or worsening their symptoms by exceeding their limits” (p. 30 [1]) and that it “is not curative” (p. 30 [1]).

As far as research evidence is concerned, a systematic review into the efficacy of pacing by Sanal-Hayes et al. concluded that “overall, studies generally reported pacing to be beneficial for people with ME/CFS”. Pacing was “the most efficacious, safe, acceptable, and preferred form of activity management for people with ME/CFS” (p. 15 [144]). “The exception to this trend is the controversial PACE trial” (p. 15 [144]). In the PACE trial, patients in the “pacing group…were…‘encouraged’ to increase physical activity levels” (p. 4 [144]) and “some view activity progression as a form of GET” (p. 17 [144]). Sanal-Hayes et al. also concluded that “the PACE trial 1-year post-intervention” follow-up showed that “pacing improved fatigue and physical functioning, with effects similar to CBT and GET” (p. 16 [144]). Moreover, as noted earlier, CBT and GET do not lead to objective improvement of fitness, and the same applies to pacing in the form of adaptive pacing therapy. Finally, according to the designers of pacing, it “is not envisaged as a treatment for the illness as a whole” (p. 1141 [145]), but it is an energy management strategy designed to “help to stabilize the condition and avoid post-exertional malaise” to try to help reduce the number and intensity of symptom flare-ups and relapses in the absence of effective (pharmacological) treatments.

As early as in 1990, microbiologist Ho-Yen proposed “a truly new approach…of moderating activity. This approach is based on experiences of 400 patients that of all treatments, rest is by far the most helpful” (p. 38 [146]). He also noted that “patients must also learn to control their activities. They must decide whether they are going to expend their energy on work, social activity, exercise or their family. They will not be able to do all of these” (p. 39 [146]). Moreover, “recognition that pursuing activity makes [the] illness worse” is important (p. 38 [146]). Consequently, microbiologist Ho-Yen was one of the first doctors to advocate for and provide evidence for the practice of pacing in 1990.

As noted earlier, White et al. support the fact that Royal Colleges are using treatment in the form of GET, which they have redefined into personalised paced exercise programmes in the absence of supportive research evidence. Yet, White et al. are opposed to it when NICE recommends it as a way of life. It also means that White et al. support the non-evidence-based use of a treatment, and the same applies to the colleges, in a time when medicine should be evidence-based.

Finally, as noted by psychology professor Hughes, “there is considerable irony in the authors’ claim that NICE have recommended a practice for which there is poor (or no) research evidence. Part of the irony is the authors’ own persistence in referring to both GET and CBT as “evidence-based” treatments for ME/CFS, even though that 356-page NICE research review showed they are not evidence-based at all” (p. 1 [137]).

## 4. Competing Interests of the Authors of White et al.

### 4.1. GRADE Evidence to Decision Frameworks and Severe Conflicts of Interest

One of the eight points raised by White et al. is that NICE used a non-standard way of applying the GRADE Evidence to Decision framework. They used two articles in support of this. The first one is the aforementioned opinion article by Turner-Stokes and Wade [130], with the latter being an author of White et al. [6]. Their second reference is an article by Alonso-Coello et al. and the GRADE Working Group [147]. This article is entitled “GRADE Evidence to Decision (EtD) frameworks: a systematic and transparent approach to making well informed healthcare choices. 1: Introduction” (p. 1 [147]). According to this article, “EtD frameworks provide an approach to structured reflection” and “can help to ensure the trustworthiness of those recommendations or decisions” (p. 9 [147]). One of the important points in that article is that “intellectual and financial conflicts of interest are common and can affect judgments and recommendations or decisions. Guideline developers and organisations responsible for healthcare decisions should consider conflicts of interest when a panel is established” (p. 3 [147]) and if there are severe conflicts of interest, then “panel members [should be excluded] from discussions of specific questions or an entire guideline” (p. 3 [147]). We mention this because there is an extensive list of competing interests at the end of the article by White et al. [6]. This list, which is 1579 words long, is almost as long as a third of the text of the article, which, including figures and boxes, is 4731 words long.

### 4.2. Undeclared Conflicts of Interest

The competing interests section does include the fact that one of their authors (Carson) “is a paid associate editor of JNNP” (p. 6 [6]), the journal their article was published in. However, a number of important conflicts of interest have not been listed. For example, another author of this article (Stone) is a member of the editorial board of the same journal. White et al. do mention that “BA [Angus] was a centre leader in the PACE trial” and Murphy “was a centre co-lead for the PACE trial” (p. 6 [6]) but they do not mention that according to the contributors section of the PACE trial, five of their other authors (Chalder, Sharpe, Wade, Wessely and White) were centre leaders in that trial too [54].

### 4.3. Undeclared Potential Financial Conflicts of Interest

White et al. also mention that another author of their article (Knoop) “reports grants from ZonMw, Stichting NKCV, MS Research, and Dutch Cancer Society, was coauthor of trials of cognitive behaviour therapy, reports royalties for a published treatment manual for CBT for fatigue in CFS/ME, and an honorarium for a lecture from Intercept Pharma Deutschland” (p. 6 [6]). They do not mention, however, that he is the director of the NKCV (the Expert Centre for Chronic Fatigue, in Dutch, het Nederlands Kenniscentrum Chronische Vermoeidheid, also known as the NKCV) [148]. The NKCV earns money from treating ME/CFS patients with CBT, which, in large part, is based on the claim of efficacy of their own studies. The NKCV is the main centre in the Netherlands that treats ME/CFS patients with CBT. The negative outcome of the updated NICE guideline might mean that Dutch healthcare insurance companies stop reimbursing CBT for ME/CFS. Consequently, Knoop also has a potential financial conflict of interest.

The competing interests section states about Chalder that she “was co-investigator of several trials of behavioural interventions for CFS/ME, including the PACE trial, has received royalties for several books and book chapters on CFS/ME and received payments for workshops on CBT for CFS/ME” (p. 6 [6]). Yet, according to the conflicts of interest section of the PACE trial, she “has done consultancy work for insurance companies” too (p. 835 [54]).

The competing interests section states about Sharpe that he “was a co-principal investigator for the PACE trial and has led a trial of CBT for CFS/ME. He is current President of the European Association of Psychosomatic Medicine Current (unpaid) and was the previous President of the Academy of Consultation Liaison Psychiatry (unpaid)” (p. 7 [6]). Yet, according to the conflicts of interest section of the PACE trial, he “has done voluntary and paid consultancy work for government and for legal and insurance companies” too (p. 835 [54]). Also, Sharpe is or was a CFS expert for Swiss Re, a reinsurance company [149].

According to the competing interests section of White et al., White “receives personal consultancy fees from Swiss Re reinsurance company” (p. 6 [6]). Yet, according to Kindlon, “if I review a Peter Denton White paper, I’ll push for him to declare he’s a CMO for a disability insurer. His current declaration doesn’t get across his major COI & how #MEcfs claims can get denied due to not doing (or not doing more) GET, if GET is seen as effective & safe” (p. 1 [150]).

According to Williams, “patients have been denied financial support from private insurers for whom Peter White and his colleagues work (for example, he was Chief Medical Officer for the giant re-insurer Swiss Re and was also CMO to Scottish Provident) and from the Department for Work and Pensions (where he was lead advisor on “CFS/ME” and was a prominent member of the group who re-wrote the chapter on “CFS/ME” in the DWP’s Disability Handbook used by Examining Medical Practitioners, by DWP decision-makers and by members of the Appeals Services Tribunals)” (p. 3 [151]).

Based on the beliefs by White, Swiss Re stated the following about CFS/ME. “CFS would fall within a mental health exclusion, if one applies to a policy. The answer to this lies within the precise exclusion wording. If the policy refers to functional somatic syndromes in addition to mental health, then CFS may fall within the exclusion. If the policy does not refer to functional somatic syndromes as well as mental health then it would be difficult to apply” (p. 1 [152]). Moreover, based on the claimed success of the PACE trial, the Swiss Re newsletter entitled “*Managing claims for chronic fatigue the active way*” states that “the key message is that pushing the limits in a therapeutic setting using well described treatment modalities is more effective in alleviating fatigue and dysfunction than staying within the limits imposed by the illness traditionally advocated by “pacing”. If a CFS patient does not gradually increase their activity, supported by an appropriate therapist, then their recovery will be slower. This seems a simple message but it is an important one as many believe that “pacing” is the most beneficial treatment” (p. 1 [152]). It also states that “funding for these CFS treatments is not expensive (in the UK, around £2000) so insurers may well want to consider funding this for the right claimants…Check that private practitioners are delivering active rehabilitation therapies, such as those described in this article, as opposed to sick role adaptation” (p. 1 [152]). Consequently, the Swiss Re newsletter also shows that fixed incremental increases are an important part of GET.

Moreover, according to the conflicts of interest section of the PACE trial, “PDW (Peter Denton White) has done voluntary and paid consultancy work for the UK Departments of Health and Work and Pensions and Swiss Re (a reinsurance company)” (p. 835 [54])

### 4.4. Undeclared Ideological Conflicts of Interest

Finally, White et al. do not mention that six of the seven worldwide leading CBT/GET proponents for ME/CFS (White, Chalder, Sharpe, Wessely, Knoop and van der Meer) are authors of their article. These researchers are deeply committed to the CB and the “aberrant” beliefs model which they have originated and/or have actively promoted for decades [12,13,14,39,54,153,154]. As a result, they are seen as leading ME/CFS experts and they have become professors. Consequently, the conclusion by NICE that both treatments do not lead to improvement or recovery undermines their careers. As noted by Dana and Loewenstein [155], physician behaviour is often influenced by self-interest; so, physicians unconsciously emphasise data that support their personal interests and “unconscious bias is a…serious problem” according to Korn and Ehringhaus [156]. Physicians and scientists are not immune to confirmation bias [157]. Like the rest of society, they are well able to ignore what they do not want to see and seek confirmation for what they believe or want to believe [156]. In view of all this, it is unclear why the authors do not declare their ideological and potential financial conflicts of interest.

## 5. Discussion

### 5.1. Review of the Evidence

In this article, we have reviewed the evidence presented by White et al., who claim that there were eight anomalies in the review process and interpretation of the evidence in the NICE guideline for ME/CFS. The main reason for their criticism is that the NICE guideline committee dropped what White et al. claim to be the only two evidence-based treatments for ME/CFS (CBT and GET), on which many of their authors have built their careers. White et al. state that “since the guideline was published, three new systematic reviews have been published” (p. 5 [6]) to support this claim. Yet, one of these three is a review of activity pacing [82]. The second one concluded that “the strength of evidence supporting the use of graded exercise and CBT was low and the magnitude of benefits was small to moderate, with inadequate evidence in patients diagnosed with more current case definitions, limited reporting of harms, and inadequate evaluation in severely affected patients” (p. iii [80]). The third one, a systematic review by Ingman et al., which included one of the authors of White et al. (Chalder), noted that “previous findings concerning short-term to medium-term follow-up are inconsistent” (p. 11 [103]). Ingman et al. also concluded that subjective outcome measures are not reliable, yet most studies used those “which may have increased the risk of observer or detection bias” (p. 11 [103]). They therefore concluded that “future trials should report objective outcomes, in addition to self-report measures” (pp. 10–11 [103]).

Another review with Chalder concluded that “work-related outcomes should be targeted in CFS treatment” (p. 6 [105]). Yet, the review itself, just like White et al., ignored those outcomes. An extensive review of the literature, however, found that CBT and GET have a negative instead of a positive effect on work and illness/disability benefit status [42].

The systematic review by Ahmed et al., which is not used by White et al., concluded that there is no evidence that CBT and GET are effective treatments for ME/CFS patients “due to methodological flaws and biases found in the studies that are investigated in this” (p. 13 [35]). Consequently, the systematic review by Ahmed et al. confirmed the conclusion by NICE that CBT and GET studies are of low or very low quality, but also that there is no evidence that those treatments are effective.

A systematic review of CBT for ME/CFS by Kuut et al. [158] was published three months after the article by White et al. was published. This review included one of the authors of White et al. (Knoop). Kuut et al. concluded that CBT is effective irrespective of the ME/CFS selection criteria used. Yet, there were many problems with this review and the studies in it, as found by a reanalysis of that systematic review [47]. For example, according to Kuut et al., the SIP8 score, which shows how disabled people are, was such that patients were still severely disabled after treatment, yet this was ignored by the review and CBT was labelled as effective. This review [158], just like White et al. [6], also ignored the absence of objective improvement [47]. According to the CBmodel, symptoms are caused by deconditioning; yet, if patients have not objectively improved, then fitness (deconditioning) has not improved, and the symptoms cannot have improved by definition of their own model. The systematic review by Kuut et al. [158], just like White et al., as we have seen earlier, also omitted potential financial and intellectual conflicts of interest [47]. For example, in Kuut et al., at least one of their reviewers was involved in every study under review. Yet, they failed to inform the readers about this. Conducting a review in this manner and not informing the readers undermines the credibility of a systematic review and its conclusion [47,158].

Six of the seven worldwide leading CBT and GET proponents are involved in White et al. (Chalder, Knoop, van der Meer, Sharpe, Wessely and White) [6]. They have all built their careers on the CBmodel and the assumption that there is no underlying disease in ME/CFS and that patients suffer from dysfunctional beliefs about the nature of their symptoms, avoidance of exercise and deconditioning. Yet, this and many other important conflicts of interest were not mentioned by White et al. [6], which undermines the credibility of their arguments.

One of the other points raised by White et al. is their disagreement of using outcomes at long-term follow-up, in general and of the PACE trial in particular. However, the three principal investigators of the PACE trial, who are all part of White et al., have repeatedly stated that the long-term follow-up results of the PACE trial showed that CBT and GET are effective. The analysis by NICE, however, showed that there was no difference between the four groups at long-term follow-up. Consequently, long-term follow-up of the PACE trial showed a null effect. According to White et al., however, NICE should have looked at the results at the end of treatment and at the six-month follow-up, even though a systematic review from 2001 had already concluded that one should look at long-term follow-up results in a chronic fluctuating disease like ME/CFS to ensure that any change is a result of the treatment and not of the natural course and fluctuation of the disease [62]. Looking at the results of the two primary outcomes at the end of treatment and six-month follow-up in the PACE trial, as requested by White et al., shows that patients were still ill enough to enter the same trial and be treated with the same treatment, as can be seen in Table 2 and Table 3. Consequently, outcomes at the preferred times by White et al. confirm the inefficacy of CBT and GET. The reanalysis of the Cochrane CBT review and the reanalysis of the amended Cochrane GET review also confirmed that [93,125]. They also found that there were many methodological problems with the CBT and GET studies for ME/CFS, as well as with both Cochrane reviews.

Moreover, as concluded by, for example, Serrat et al. in 2023, “no single therapeutic approach supported by a RCT has shown to be definitely effective, with coherence and reproducibility, for CFS/ME management. Treatment options are limited, which represents a significant unmet medical need. Indeed, methodological reviews have criticised the scientific rigor of previous RCTs regarding the effectiveness of CBT and therapeutic exercise for CFS/ME” (p. 2 [159]). They themselves conducted a trial into the efficacy of an online multicomponent intervention called FATIGUEWALK for ME/CFS. They concluded that their treatment seemed effective but to find out if it really is, “further trials, including active control groups with an equivalent treatment dose” are needed (p. 1 [159]). Almost all of the studies used by White et al. in support of their claim of efficacy of CBT and GET did not use active control groups with an equivalent treatment dose. As we saw earlier, setting up a non-blinded study using a badly designed non-active control group and subjective outcome measures is the way to prove that your therapy is effective, even when it is not according to psychology professor Cuijpers [71]. The influence of a non-active control group on the outcome of a study was highlighted by a systematic review of CBT for many different conditions, including ME/CFS. Fordham et al. concluded that the effect size was 0.31 if the studies used a non-active comparator control group and 0.09 when an active comparator control group was used [160]. A score between 0.20 ≤ and <0.50 signifies that the effect of a treatment is small, and a score below 0.20 that it is ignorable [161,162]. Consequently, using a poorly designed (non-active) control group instead of a properly designed active control group artificially inflated the results of the CBT and of the GET studies for ME/CFS.

Using badly designed control groups and subjective outcomes in non-blinded studies is even more of a problem because one of the other important problems with CBT and psychotherapy studies is the allegiance of the researchers to the treatment. “Allegiance in psychotherapy represents the therapist’s personal belief both in the superiority and the efficacy of a particular treatment” (p. 1 [163]). A systematic review by Dragioti et al. concluded that “experimenter’s allegiance influences the effect sizes of psychotherapy RCTs and can be considered non-financial conflict of interest introducing a form of optimism bias, especially since blinding is problematic in this kind of research” (p. 1 [163]). This is of particular interest because many researchers of White et al. [6], for example, White, Angus, Buchwald, Chalder, David, Fink, Flottorp, Henningsen, Knoop, Lloyd, van der Meer, Miller, Sharpe, Wessely and Wyller, have built careers on the CBmodel and the efficacy of CBT (and GET) for ME/CFS.

Finally, why White et al. continue to rely on subjective outcomes in their non-blinded studies that use badly designed control groups to claim that their treatments are effective is unclear, especially in light of the fact that, according to the GES Therapists Manual by White himself, “the sense of effort is not a reliable indication of physiological effort in a patient with CFS/ME. So distance covered in a specific time can replace this, and should be used at the stage of gradually increasing the intensity of exercise/activity” (p. 48 [164]). In the article of that study, the authors conclude that subjective outcomes are unreliable when they state that “we did not measure any objective outcomes, such as actigraphy, which might have tested the validity of our self-rated measures of physical activity” (p. 10 [57]). Studies that used objective outcomes show that CBT and GET do not lead to objective improvement, as can be seen in Table 5. Moreover, both treatments have a negative instead of a positive effect on work and benefits status [42].

Why White et al. ignore that White himself concluded that subjective outcomes are not reliable in ME/CFS is unclear. Even more so because the unreliability of subjective outcomes in non-blinded CBT and GET studies for ME/CFS was highlighted by the amended Cochrane exercise review from 2020 [93], as we saw earlier. Why they also ignore the recommendation that the patient’s perspective should be given the most weight, when using subjective outcome measures because these are patient-related outcome measures [165], is unclear. It is also unclear why White et al. ignore that, according to their own PACE trial, CBT and GET do not lead to an improvement in CFS symptom count. Consequently, and as concluded by a systematic review by Ahmed et al. that is not used by White et al. [6], “the findings of this systematic review do not support the claim that CBT and GET are effective treatments for ME/CFS patients” (p. 13 [35]).

### 5.2. Redefining the Disease

One of the other anomalies according to White et al. [6] is the fact that NICE makes PEM compulsory for diagnosis and, by doing so, they claim that NICE has redefined the disease. However, the NICE guideline from 2007 [53] already stated that if patients do not suffer from PEM, then the diagnosis needs to be reconsidered. Why White et al. [6] are not aware of that is unclear because two of their authors (Hamilton and Santhouse) sat on the committee that created that guideline [53]. Moreover, the name ME goes back to an outbreak in a London hospital in 1955. The infectious disease specialist of that hospital noted that the main characteristic of the disease was an abnormally delayed muscle recovery after trivial exertion, which over the years, evolved into PEM [30]. In 1991, a group of British doctors created the Oxford criteria, and they erased the aforementioned main characteristic of the disease and replaced it with severe chronic disabling fatigue. In 1994, a group of Americans, with some international representation, created the Fukuda criteria and PEM became only an optional criterion. Respectively, five (Oxford criteria) and three authors (Fukuda criteria) of White et al. [6] were involved in removing PEM as a compulsory criterion for the diagnosis of ME/CFS in 1991 and 1994 [32,33]. Consequently, the disease was redefined twice involving authors of White et al., as long ago as in the 1990s, and not by NICE. It also means that redefining the disease by authors of White et al. has not only confounded their own research, but it also plays an important part in the fact that there are no effective treatments for post-infectious diseases like ME/CFS and long COVID. This is even more of a problem because, according to a conservative estimate, at least 400 million people are suffering from long COVID [7]. According to a systematic review, 51% of long COVID patients fulfil ME/CFS criteria that include a mandatory PEM symptom [8]. Consequently, COVID-19 has led to more than 200 million extra ME/CFS patients, in addition to the 17 to 24 million that were already afflicted according to a recent meta-analysis by Lim et al. [9]. However, as noted in a recent letter by Vardaman and Gilmour [166], the cited reference for this by Lim et al. is not about ME/CFS but it is about the global burden of multiple sclerosis which is not relevant for ME/CFS. Vardaman and Gilmour [166] also noted that Lim et al. reported an estimated prevalence of 0.89%. A meta-analysis by Johnston et al. [167] found that the pooled prevalence was 0.76% for clinically assessed ME/CFS. Both meta-analyses used the Fukuda criteria. However, PEM, the main characteristic of the disease, is not compulsory for diagnosis according to these criteria as we have seen earlier. Research found that 15% of people labelled by the Fukuda criteria as having ME/CFS, were in fact healthy people [93]. Taking this into account would mean that there are an estimated 51.7 (0.76% × 85% × 8B), respectively 60.5 (0.89% × 85% × 8B) million people with ME/CFS, based on a world population of 8 billion people (= 8B) [166], and not 17 to 24 million [9]. Consequently, there are an estimated 251.7 to 260.5 million people with ME/CFS worldwide and up to 75% of them are unable to work according to an extensive review of the literature [42]. This has an enormous impact on the world’s economy [10] and the medical world is currently clutching at straws on how to treat patients with post-infectious diseases. Many doctors and other healthcare professionals are now starting to realise how debilitating these diseases are. Research shows that ME/CFS and long COVID share many symptoms and there are many clinical similarities. It also shows that there are many underlying biological abnormalities reported in both illnesses, documented by many different laboratories [168]. ME/CFS typically has a longer disease duration than long COVID, with symptoms and pathologies evolving over time. Consequently, ME/CFS research may provide insights into the shared disease pathologies and the future progression of long COVID. Understanding these disease pathologies will be key to developing effective therapeutics and diagnostic tests, according to a recent review by Annesley et al. [169].

### 5.3. Quality of Life

One of the authors of White et al. (Wessely) criticised NICE in July 2021 in the Stakeholder position statement on the NICE guideline for depression in adults by stating the following: “NICE must run a reanalysis of studies using quality of life and/or functioning outcomes where these are available and prioritise recommendations based on these measures, given that these are the measures of greatest priority to service users” (p. 5 [61]). Most CBT and GET studies for ME/CFS do not use quality-of-life scores as an outcome measure. Exemptions are the biggest CBT study from the Netherlands by Prins et al. [107], which found that CBT was not more effective than the natural course (no treatment) for quality of life. Jason et al. [94] found that quality-of-life scores improved 5 per cent more after relaxation than after CBT. Group CBT did not bring about an improvement in quality of life in O’Dowd et al., as found by a re-analysis of the Cochrane CBT review [125,170]. The PACE trial found that the quality-of-life scores (EQ-5D) at 24 weeks (end of treatment) of 0.60 (GET) and 0.61 (CBT) and of 0.59 (GET) and 0.63 (CBT) at 52 weeks (six-month follow-up) [115] were similar to the score (0.60) for people with five or more chronic health conditions with the exception of the CBT score at 52 weeks. Those scores were also worse or similar to the score in cerebral thrombosis (0.62). All four scores were worse than in angina and rheumatoid arthritis (0.65), acute myocardial infarction (0.66) [171], MS (0.67), lung cancer (0.69), stroke (0.71) or ischaemic heart disease (0.72) (linear scale ranging from –0.624 to 1.000, where negative values are conditions considered worse than death; higher scores indicate a better quality of life) [172].

As far as functioning outcomes are concerned, the PACE trial found that CBT and GET did not have an effect on CFS symptom count. The aforementioned systematic review of CBT for ME/CFS, by one of the authors of White et al. (Knoop) [158], ignored that patients remain severely disabled after treatment with CBT according to their own SIP8 figures, as found by a re-analysis [47]. As can be seen in Table 5, both treatments do not lead to objective improvement and an extensive review of the literature found that CBT and GET have a negative instead of a positive effect on work and illness and benefit status [42]. Consequently, if NICE would have prioritised its recommendations based on the quality of life and functioning outcomes, given that these are the measures of greatest priority to service users according to Wessely, one of the influential authors of White et al. [6], then these outcome measures would confirm the conclusion by NICE that CBT and GET are ineffective treatments which should not be recommended. At the same time, it is important to realise that the earlier discussed unreliability of relying on subjective outcomes in non-blinded trials also applies to the quality of life and subjective functioning outcomes. However, it is likely that these two subjective outcomes are less unreliable in this case because their null effect is in line with the null effect of the objective outcome measures.

### 5.4. Are There Fixed Increments in GET?

White et al. also claim that it was incorrect that NICE states that GET is harmful and should not be used because incremental increases are an essential part of it, whereas according to White et al., “there are no ‘fixed increments of exercise’ in GET” (p. 5 [6]). They also claim that “we have been unable to find any trials that prescribed fixed increments of exercise” (p. 4 [6]). Our analysis, however, showed that the GET manual for therapists (final version 7) of the PACE trial [140], for example, shows that fixed incremental increases are an essential part of GET. This manual states, for example, that “The aim of this treatment is to reverse the physical inactivity that helps to maintain CFS/ME” (p. 20 [140]) but also that “a central concept of GET is to MAINTAIN [sic] exercise as much as possible during a CFS/ME setback” (p. 51 [140]). This manual also states that “GET involves the…gradual and planned increases in physical activity or exercise” and to ensure that the therapist truly understands that, this is followed by “it is their *planned physical activity, and not their symptoms* [italic by us], that determine what they are asked to do” (p. 20 [140]).

Why White et al. are unaware of this is unclear because this manual was written by White, who is the leading author of White et al. [6]. Also, eight authors of White et al. were involved with the PACE trial [6,54]. Three of them as principal investigators and as centre leaders (Chalder, Sharpe and White), four of them as centre leaders (Angus, Murphy, Wade and Wessely) and one (Miller) as one of the three independent assessors of the trial’s safety data [54]. That GET is not symptom-dependent is also made clear by other PACE trial manuals, the GET study by Moss-Morris et al. and a newsletter from an insurance company for which Professor White works or has worked [95,152]. Why this is unknown to White et al., and why they could not find this, is unclear.

Our analysis also shows that fixed incremental increases are part of CBT for ME/CFS as well. Consequently, NICE should also have concluded that because of that, CBT is not safe to be used either and therefore should not be used.

### 5.5. Why GET Is Harmful

#### 5.5.1. A Graded Increase in Exercise

According to White et al., GET is not harmful because “systematic studies of the safety of GET found no convincing evidence of harm with GET” (p. 4 [6]). Yet, the deleterious effect of physical exercise was highlighted as early as in 1978 by a review of the ME outbreaks over the years [173]. In 1989, Wessely et al. proposed to manage ME/CFS by treating the “avoidance behaviour” to enable “a gradual return to normal physical activity” (p. 26 [12]). In that study, the patients were treated with a behavioural programme which involved a number of things, for example, “a graded increase in exercise” with a “gradual exposure to all avoided activity” in combination with “cognitive work to break the association between increase in symptoms and stopping or avoiding the activity” (p. 27 [12]). Microbiologist Ho-Yen [146] responded to this and questioned the safety of a gradual increase in exercise. After reviewing 400 patients, he concluded that “exercise” and “pursuing activity makes [the] illness worse” (p. 38 [146]). Wessely et al., however, dismissed Ho-Yen’s concerns by stating that “it is difficult to think of a pathological mechanism by which gradual increased activity could be harmful” (p. 83 [174]). The references at the end of that sentence refer to two articles about neuromuscular disease. The first one is a theoretical article [175] about rehabilitation in hereditary myopathies, especially in progressive muscular dystrophy. The second one is a study [176] that investigated the effects of weight training on muscle performance in 16 patients with gradually progressive neuromuscular disorders. Why Wessely et al. used those two articles to dismiss the concerns by the microbiologist is unclear because ME/CFS is not a progressive neuromuscular disorder/a hereditary myopathy. It is also unclear why the four authors of the article by Wessely et al., three of which are also authors of White et al. (Chalder, David and Wessely) [6], who are all mental health specialists, dismissed the concerns of the microbiologist without investigating them.

#### 5.5.2. Wanting to Rely on What Patients Say

White et al. claim that they want to rely on what patients say but they dismiss the voice of patients who have repeatedly stated, since 1990, that CBT and GET are not only ineffective but also very harmful, as highlighted in Table 4 by a number of patient surveys. Why they dismiss that is unclear, especially in light of the fact that they use an article about making well-informed healthcare choices. According to this article by Alonso-Coello et al., important questions that need to be asked to come to well-informed healthcare choices include questions about the certainty of the evidence and about the benefits and harms of a treatment, but it should also be considered “how patients (or others affected, such as carers) value the main outcomes” (p. 3 [147]). This is even more important because, as we saw earlier, 32% of newly approved treatments are found to be harmful when they are used in real life [75]. Especially affected are psychiatric treatments, and harmful effects of the treatments are usually brought to the attention of the medical profession by patients. Moreover, as concluded by Ernst and Pittler, “systematic reviews and meta-analyses provide little information on the safety aspects of therapeutic interventions”. And “the assessment of safety has to go far beyond randomised clinical trials” because studies “typically assess only a small number of patients” and they “are usually of short duration and thus cannot identify delayed adverse events” (p. 546 [177]).

#### 5.5.3. A Systematic Review by White and Etherington

White et al. use a systematic review in support of their claim that GET is safe. However, they do not mention that this review was carried out by two authors of White et al. (White and Etherington) [76]. They also do not mention that one of the problems with that review was that their definition of harm did not include everybody who was negatively affected by the treatment, nor do they mention that, for example, the amended Cochrane exercise review concluded that it is uncertain if GET is safe or not “because the certainty of the evidence is very low” and because most studies did not report about safety (p. 37 [78]), thereby confirming the above-mentioned conclusion by Ernst and Pittler that systematic reviews provide little information on the safety aspect of therapeutic interventions [177]. Why this is ignored by White et al. is unclear because two of its authors were involved in that Cochrane review [78,79]. Moreover, in White’s own GETSET trial, which was one of the 10 studies in the systematic review by White and Etherington, physiotherapist-rated data on adherence to GES was available for 97% of participants who were offered GES. These data showed that only 42% adhered to GES completely or very well, 30% adhered to it moderately and 29% slightly or not at all (p. 8 [57]). Consequently, the majority (59%) did not adhere (very well) to the treatment. As noted earlier and concluded by psychologist professor Lilienfeld, clients who dropped out may not have been helped or may even have been harmed by the intervention [83].

#### 5.5.4. The GET Participants Manual of the PACE Trial

According to the GET participants manual of the PACE trial, “GET is the use of regular, physical exercise” with a gradual increase in an individual’s level of activity “to aid recovery from CFS/ME” (p. 107 [178]). When patients claim that that leads to flare-ups and relapses, then that is incorrect according to this manual because “the evidence we have is in fact the opposite: there is no evidence to suggest that an increase in symptoms is causing you harm. It is certainly uncomfortable and unpleasant, but not harmful. In fact, there is much evidence to support the alternate view: if you rest too much, it is the resting that can cause negative changes in the body. Resting and withdrawing from activity can also make us feel fed up or worried, and this can also make it harder to continue being active” (p. 79 [178]). That manual also states that “setbacks are a normal part of recovery” (p. 81 [178]), which “are likely to become less severe and last for less time than previously as I get stronger”, but also that “I should try to keep to my physical activity or exercise plan as much as possible, in order to maintain my physical health during this time”. In addition, the manual states that “resting too much may feel like the right thing to do now, but in the long run is likely to worsen my condition” (pp. 81–82 [178]). And “if you are having a very severe setback, in which your previous level of activity is leading to distressing or unmanageable symptoms, then *reducing activity slightly* [italic by us] and increasing rest might be a temporary solution, as long as you build up again as soon as you can to your previous level. Building up again should ideally occur within a few days to avoid the detrimental effects of rest. Reducing activity should be avoided if at all possible” (p. 79 [178]). Yet, following that advice will turn PEM and flare-ups into severe relapses and that is an important reason why 80% of patients found GET to be harmful, but also, why 22% of patients were made bedridden by GET according to research by the Oxford Brookes University [89].

#### 5.5.5. The IOC’s 10% Rule

Another important reason why GET might be harmful is the following. According to the GET participant and therapist manuals of the PACE trial, after negotiating and establishing a baseline of low intensity exercise, then one should “add 20% duration, up to 30 min” followed by a “gradual increase in intensity up to target heart rate” (p. 38 [140] and p. 32 [178]). According to the GES therapist manual of the GETSET trial, “progress is recommended as 20% increases in duration or intensity once a baseline has been set” (p. 47 [164]). In Moss-Morris et al., the participants had to start with “10–15 min [of exercise] 4 to 5 times a week” (p. 249 [95]), followed by “increases generally involved duration increases of three to five minutes per week” and “the final goal was for each participant to be exercising for approximately 30 min for 5 days per week” (p. 249 [95]). Consequently, the participants had to increase their weekly training load by 30–33%. Yet, according to the consensus statement on load in sport and risk of injury of the International Olympic Committee (IOC), “athletes should limit weekly increases of their training load to <10%” and they should do that “to stay in positive adaptation and thus reduce the risk of injuries” (p. 1036 [179]). Consequently, as can be seen in Table 6, in the PACE Trial, in the GETSET trial and in Moss-Morris et al., the training load imposed on ME/CFS patients by GET is such that it increases the risk of injuries, which is another reason why GET is potentially harmful.

#### 5.5.6. Additional Reasons Why GET Is Harmful

Other reasons why GET is harmful include telling patients:

- That “there is nothing to stop your body from gaining strength and fitness” (p. 31 [178]), whereas there is an underlying illness preventing that [1,2].
- That if patients suffer a flare-up, they need to “try to keep to your exercise and activity plan, knowing that in time your body will adjust” (p. 79 [178]), whereas if patients would do that, that would turn a flare-up into a relapse and a relapse into a severe relapse.
- That if patients “are having a very severe setback” with “unmanageable symptoms, then reducing activity slightly and increasing rest might be a temporary solution” (p. 79 [178]), whereas in the absence of effective treatment, trying to continue to exercise during a very severe setback, will render patients bedridden for life.
- “that despite a setback, creating a ‘dip’…the overall trend [if they continue to exercise] is usually upwards” (p. 77 [178]), whereas if patients continue exercising during a setback, then they will go over their boundaries again, causing severe flare-ups and relapses.
- That exercise will not cause them harm, yet there are “many negative consequences of rest” (p. 26 [164]), even though rest is the only thing that might reduce the damage caused by exercising.
- That “setbacks are a normal part of CFS/ME recovery” and not a worsening of the disease and that these symptom flare-ups “are likely to become less severe and last for less time than previously as I get stronger” (p. 81 [178]); yet, if patients do that, then flare-ups become much worse and turn into severe relapses.
- That “activity or exercise cannot harm you” (p. 49 [180]), whereas research (discussed below) highlights the harmfulness of exercise in ME/CFS [17,18,19,20,21,22,23,24,25,26,27,28,29].
- That “medical research evidence shows … [that there is] no underlying serious disease” (p. 37 [180]) but also that “there is no evidence to suggest that an increase in symptoms is causing you harm. It is certainly uncomfortable and unpleasant, but not harmful” (p. 79 [178]). Yet, as concluded after an extensive review of the literature, by the Institute of Medicine, ME/CFS is a chronic and debilitating multisystem disease and not a psychological or psychosomatic one. The IOM, the Dutch health Council, the Superior Health Council of Belgium, NICE and the German IQWiG Institute came to the same conclusion [1,2,3,4,5].
- That “a central concept of GET is to MAINTAIN [sic] exercise as much as possible during a CFS/ME setback” (p. 51 [140]). Yet, as mentioned above, that will turn setbacks into severe relapses and render patients bedridden for life.
- That “due to greater levels of inactivity in the more severely disabled group, the deconditioning model should apply equally if not more to these patients” (p. 24 [140]). Yet, many of the more severely disabled have become bedridden as a consequence of being treated with GET.
- That “athletes tell us that when they exercise hard, they also get muscle soreness as a result of challenging their muscles. We believe that this symptom of CFS/ME is a normal response to increased exercise or physical activity, and that it can even be seen as a positive sign that our body is being challenged and is strengthening” (p. 162 [140]). Yet, in reality, muscle soreness is not the problem. The real problem in this case is severe muscle pain, the myalgia part in myalgic encephalomyelitis, as a consequence of the exercise program. This is not a normal response to exercise, nor is it a positive sign that the patients’ bodies are strengthening. Instead, it is a sign of a bad flare-up or a bad and severe relapse.

In the absence of effective pharmacological treatment, there is no way to combat the above-mentioned problems other than resting to try and prevent PEM, flare-ups and relapses. Moreover, rest is also the only thing that might help during flare-ups and relapses.

#### 5.5.7. The Effects of Exercise in ME/CFS

Many studies have provided objective evidence for the harmfulness of exercise in ME/CFS. Black and McCully, for example, concluded “that CFS patients may develop exercise intolerance...after 4–10 days. The inability to sustain target activity levels, associated with pronounced worsening of symptomology, suggests the subjects with CFS had reached their activity limit” (p. 1 [17]). Kujawski et al. looked at the effects of exercise in ME/CFS and concluded that “exercise was not well tolerated by 51% of patients” (p. 1 [19]). Lien et al. [18] found that physical performance deteriorates and lactate levels increase in response to exercise in patients with ME/CFS, whereas the exact opposite happens in healthy people. Moreover, many studies have provided objective evidence for exertional intolerance, delayed muscle recovery and other physical abnormalities in ME/CFS following exercise, which were not seen in the healthy (sedentary) controls [17,18,19,20,21,22,23,24,25,26,27,28,29].

The very high dropout rates after 6, 9 and 12 months of 55%, 73% and 80%, respectively, as found by an analysis of the efficacy of GET in the sports medical department of a Dutch hospital [40,181], highlight the inappropriateness of GET for ME/CFS, even in only very mildly affected patients and even when it is tailored to the patient and carried out at 50 to 60% of the VO2max. Given all these considerations, one cannot conclude that GET is safe. The safety of patients should always come first, and if one does not know if a treatment is safe or, as in this case, there is ample evidence that GET is harmful, then GET should not be used, promoted or recommended.

## 6. Conclusions

Our analysis shows that the arguments that are used to claim that there are eight anomalies in the review process and the interpretation of the evidence by NICE are anomalous and they highlight the absence of evidence for the claims that are made. Our analysis not only exposes the fixed incremental nature of GET, but also of CBT, for ME/CFS.

## Figures and Tables

**Table 1:** Fatigue and physical functioning scores at the end of treatment in the GETSET trial.

Trial	Likert Fatigue Score	Physical Functioning
GETSET trial score at 12 weeks (end of treatment) [57].	19.1	55.7
PACE trial entry requirement [54].	18 or more	65 or less
Severely ill	More than 9 (= bimodal score of more than 3) [58].	70 or less [59].

**Table 2:** Likert fatigue scores after treatment with CBT and GET in the PACE trial [54].

Fatigue Scores	24 Weeks (End of Treatment)	52 Weeks (6-Month FU)
CBT	21.5	20.3
GET	21.7	20.6
Fatigue entry requirement	18 or more	18 or more
Fatigue recovery score according to the PACE trial protocol [72].	6 to 9 or less (= bimodal score of 3 or less)	6 to 9 or less (= bimodal score of 3 or less)
Severely ill [58].	More than 9 (= bimodal score of more than 3)	More than 9 (= bimodal score of more than 3)

FU: follow up.

**Table 3:** Physical functioning scores after treatment with CBT and GET in the PACE trial [54].

Physical Functioning	24 Weeks (End of Treatment)	52 Weeks (6-Month FU)
CBT	54.2	58.2
GET	55.4	57.7
Physical functioning entry requirement	65 or less	65 or less
Severely ill [59].	70 or less	70 or less
Physical functioning recovery score according to the protocol [72].	85 or more	85 or more

FU: follow-up.

**Table 4:** Patient surveys by ME Action/Action for ME.

Patient Surveys	Sample Size	Safety of CBT and GET
ME Action (1990)	*n* = 695	GET harmful in 49.6%
Action for ME (2001)	*n* = 2338	GET harmful in 50%; CBT harmful in 26%
Action for ME (2007)	*n* = 332	GET harmful in 74%; CBT harmful in 18%
Action for ME together with Action for young people with ME (2008)	*n* = 2763	GET harmful in 34%; CBT harmful in 12%
Action for ME (2011)	*n* = 273	GET harmful in 60.2%
Action for ME (2014)	*n* = 1161	GET harmful in 47%; CBT harmful in 12%

Source: ME Action (1990), Action for ME (2001) and Action for ME together with Action for young people with ME (2008): [85]. Action for ME (2011): [88]. Action for ME (2007) and Action for ME (2014): [88].

**Table 5:** Results of objective outcomes in CBT and GET studies.

Trials	Rx	N	Active Control Group	Objective Outcome	Objective Improvement?	Objective Null Effect Reflected in Trial’s Conclusion?
Prins et al. (2001) [107].	CBT	278	No	Actometer, work status, objective neuropsychological tests	No	No
Moss–Morris et al. (2005) [95].	GET	49	No	VO2 peak	No	No
Stulemeijer et al. (2005) [108,109].	CBT	71	No	Actometer	No	No
Knoop et al. (2007) [110].	CBT	233	No	Objective neuropsychological tests *	No	No
Knoop et al. (2008) [109,111].	CBT	171	No	Actometer	No	No
Stordeur et al. (2008) [112].	Evaluation of the efficacy of CBT and GET in Belgium CFS centres	655	Evaluation study, no control group	Exercise test	No	No
Wearden et al. (2010 and 2013) [113,114].	CBT and GET	296	No	Timed step test	No	No
White et al. (2011) [54,115,116].	CBT and GET	641	No	6MWT; step test; Work and illness/disability benefit status.	No	No
Nijhof et al. (2012) [117,118].	Internet-based CBT	135	No	Actometer	No	No
Vos-Vromans et al. (2017) [119].	MRT **	122	CBT	Actometer	No	No

*: The objective neuropsychological tests in Prins et al. (2001) [107] were reported in Knoop et al. (2007) [110] and consisted of two reaction time tests and a symbol digit modalities task; these are actometer results, just like those from Stulemeijer et al. (2005) [108] and Knoop et al. (2008) [111], published by Wiborg et al. in 2010 [109]; **: MRT: multidisciplinary rehabilitation treatment which includes CBT.

**Table 6:** Weekly increase in training load in GET studies.

Study	Weekly Increase in Training Load
The PACE trial [140,178]	20%
The GETSET trial [164]	20%
Moss-Morris et al. [95]	30–33%
Maximum weekly increase to reduce the risk of injuries according to the IOC [179]	<10%

## Data Availability

Not applicable.
